# A Highly Sensitive and Selective Probe for the Colorimetric Detection of Mn(II) Based on the Antioxidative Selenium and Nitrogen Co-Doped Carbon Quantum Dots and ABTS^•+^

**DOI:** 10.3389/fchem.2021.658105

**Published:** 2021-07-02

**Authors:** Qinhai Xu, Xiaolin Liu, Yanglin Jiang, Peng Wang

**Affiliations:** Department of Chemistry, Renmin University of China, Beijing, China

**Keywords:** carbon quantum dots, ABTS^•+^, colorimetric, SE, Mn(II)

## Abstract

Herein, selenium and nitrogen co-doped carbon quantum dots (Se/N-CQDs) were hydrothermally synthesized by using citric acid, histidine, and sodium selenite, which had sp^3^ and sp^2^ hybridized carbon atoms and showed excitation-dependent fluorescence behavior. Furthermore, due to the redox reaction of ABTS^•+^ and Se/N-CQDs, Se/N-CQDs had the excellent antioxidant capacity that it was demonstrated by scavenging ABTS^•+^ with the fading of blue. Based on the synergistic effect of Se/N-CQDs and Mn(II) on ABTS^•+^, Se/N-CQDs and ABTS^•+^, as a stable, sensitive, selective, and reproducible colorimetric sensor, was applied to the detection of Mn(II) with a detection limit of 1.69 μM and a linear range of 0 to 142.90 μM. More importantly, the probe was successfully applied to detecting Mn(II) in tap water, illustrating that it could be a promising tool for Mn(II) detection in water environments.

## Introduction

Metal ions play important role in various biological, chemical, and environmental processes (Swami et al., 2018). For example, Mn(II) is a necessary trace element in the human body because of the important role it plays in bone mineralization, protein and energy metabolism, and cellular protection from injurious free radical kinds (Shokrollahi and Shokrollahi, 2014). However, an abnormal level of Mn^2+^ could bring about nervous system disorders, dermatitis, mitochondrial abnormalities, etc. (Zhou et al., 2012; Shokrollahi and Shokrollahi, 2014). Thereby, monitoring the presence of Mn(II) in water is of general interest in order to maintain both our health and the safety of our drinking water (Fukushima and Aikawa, 2020). Conventional analytical instruments include atomic absorption spectrometry (AAS), inductively coupled plasma optical emission spectrometry (ICP-OES), electrochemistry (EC), fluorescence (FL), and colorimetry (CS) (Narayanan and Park, 2014; Narayanan and Han, 2017; Zhang et al., 2018). Among them, colorimetry (CS) as a rapid, simple, and easy processing method has also been of interest in recent years. Some colorimetric probes are effective methods for Mn(II) ion detection (Kim et al., 2014; Yi and Zhang, 2016; Han et al., 2017). Recently, Yasumasa Fukushima et al. reported a colorimetric sensor based on a mixture of an anionic pyridylazo dye and a cationic polyelectrolyte for the detection of Mn(II) in the range of 0–9 μM and the detection limit of 2.23 μM (Fukushima and Aikawa, 2020). Bhamore et al. synthesized glutathione-capped syzygium cumini carbon dot amalgamated agarose hydrogel film for naked-eye detection of Mn(II) with the concentration ranges of 0.0075–0.1 mM and the detection limit of 2.1 μM (Bhamore et al., 2020). Van-Tuan Hoang et al. used the functionalized-AgNPs in the detection of Mn(II) over a range of 0–100 mM and the limit of detection of 0.22 mM (Hoang et al., 2020). Jianyu Wei et al. developed a novel tridentate ligand based on AgNPs for the colorimetric detection of Mn(II) over the range of 0.05–10 μM and the LOD of 12.6 nM (Wei J. et al., 2018). Memoona Najeeb et al. developed a simple colorimetric method based on the catalytic oxidation ability of silver nanoparticles for the detection of Mn(II) in the concentration range of 0.1–5 μM and a detection limit of 52 nM (Najeeb et al., 2018). Nevertheless, in order to get better detection performances, it is still important to develop a simple, sensitive, and selective colorimetric probe for the detection of Mn(II) ions.

Carbon quantum dots (CQDs), a kind of carbon nanomaterial with a size below 10 nm, have drawn increasing attention because of the excellent solubility, high stability, biocompatibility, low toxicity, and excellent fluorescence properties (Zhang and Chen, 2014; Liu et al., 2017). The hydrothermal carbonization (HTC) process as a simple and economic method has been widely applied to synthesize CQDs in recent years (Titirici and Antonietti, 2010; Li et al., 2012). Currently, CQDs, as a colorimetric sensor, have been applied to detect different ions (Wang and Hu, 2014; Baruah and Chowdhury, 2016; Narayanan and Han, 2017; Zhang et al., 2018). In addition, CQDs as antioxidants have been attracted attention by scavenging free radicals (Sachdev and Gopinath, 2015; Chunduri et al., 2016; Shen et al., 2017), which have the advantage of good antioxidant activity, excellent water stability and solubility, high quantum yield, stable photoluminescence, easy surface functionalization, excellent biocompatibility, low-cost, and negligible toxicity (Wang et al., 2019; Mahat and Shamsudin, 2020; Surendran et al., 2020). The action principles of CQDs as antioxidants for free radical scavenging could be attributed to the large number of carboxyl and hydroxyl groups present on the surface (Chunduri et al., 2016; Wang et al., 2019), adduct formation at the sp^2^ sites of the carbon network (Wang et al., 2019), heteroatom (N, S, Cl) doping (Zou et al., 2019; Markovi et al., 2020). What's more, due to the synergetic effect of multiple heteroatoms doping in the carbon lattice (Wang et al., 2015; Liu et al., 2020; Tripathi et al., 2020), the multielement (like N, Se, B, S, and P) co-doped CQDs have attracted much attention because of their novel properties different from single heteroatom doped CQDs and simple CQDs (Ruiqi et al., 2018; Li et al., 2019). Co-doped CQDs with different heteroatoms could afford more active sites and improve the fluorescent quantum yield (Ruiqi et al., 2018; Wei J. M. et al., 2018), tuning the photophysical characteristics of CQDs (Zhu et al., 2020), increasing electrical conductivity and specific surface area (Rahbar et al., 2019), obtaining dual-functional mode properties (Mohammed and Omer, 2020), and enhancing the properties of antioxidant capacity, analysis, detection, catalytic, electronic, and optical applications (Samantara et al., 2015; Li et al., 2016; Liu et al., 2019). Among the elements, the doping of nitrogen (N) into CQDs (N-CQDs) could not only improve the quantum yield and fluorescence efficiency but also provide active sites in the CQDs and impart unexpected surface properties to broaden the potential applications in the antioxidation, analysis, catalysis, and so on (Niu et al., 2015; Deng et al., 2018; Lu et al., 2018; Atchudan et al., 2019; Qi et al., 2019). Selenium plays an important role in a range of biological processes through its crucial function in antioxidant defense (Li et al., 2017). Selenium doped carbon quantum dots (Se-CQDs) were synthesized and applied as antioxidants and sensors (Yang et al., 2015; Li et al., 2017). Furthermore, the measurement of radical scavenging activity of antioxidants is commonly associated with the use of the ABTS^•+^ method because it is a quick, reliable, and reproducible method (Srihari and Satyanarayana, 2012). However, less attention has been paid to the application of selenium/nitrogen-doped carbon quantum dots (Se/N-CQDs) and ABTS^•+^ (Wu et al., 2013; Sun et al., 2018). As a colorimetric sensor for the Mn(II) detection, Se/N-CQDs and ABTS^•+^ have not been reported in the literature for the moment. Accordingly, synthesizing Se/N-CQDs as antioxidants and Mn(II) sensors is significant work.

**Scheme 1:**
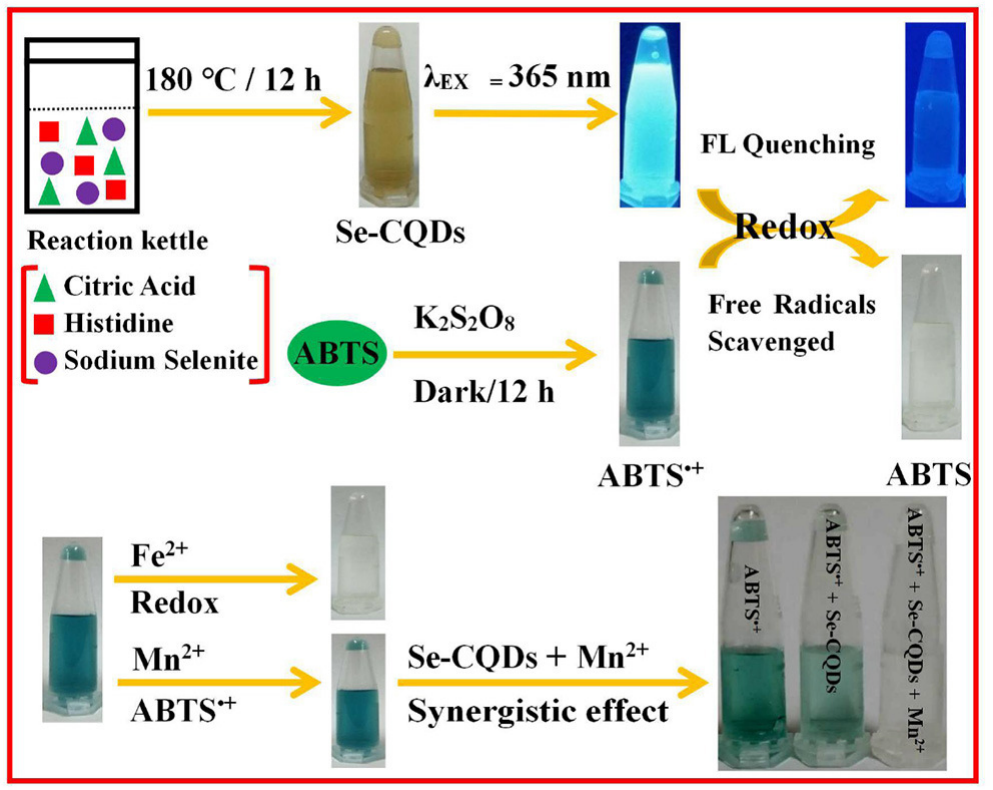
The preparation of Se-CQDs and ABTS^•+^ and the colorimetric detection of Mn(II) by Se-CQDs + ABTS^•+^.

Herein, a hydrothermal method was used to prepare Se/N-CQDs by using citric acid and histidine as carbon and nitrogen sources and sodium selenite as a selenium source. Se/N-CQDs emitted the blue fluorescence with a crystalline carbon structure. More importantly, the redox reaction could have existed between Se/N-CQDs and ABTS^•+^ with the fading of blue. The radical scavenging activity of Se/N-CQDs was greater than that of Trolox by scavenging ABTS^•+^, exhibiting that Se/N-CQDs possessed good antioxidant capacity. Moreover, due to the synergistic effect of Se/N-CQDs and Mn(II) on ABTS^•+^, ABTS^•+^ and Se/N-CQDs, as an excellent colorimetric sensor, were applied a determinate concentration of Mn(II) (Scheme 1). The probe was also applied to the detection of Mn(II) in a real sample.

## Materials and Methods

### Reagents and Apparatus

All the needed reagents and apparatus in the studies are shown in Supplementary Material.

### Preparation of Se/N-CQDs

A total of 0.5 g of citric acid, 0.4 g of histidine, and 0.4 g of sodium selenite were dissolved in 30 mL ultra-pure water. The mixed solutions were transferred in to a 50 mL autoclave and heated at 180°C for 12 h. The product was centrifuged at 15,000 rpm for 10 min, filtered with 0.22 μm micro filtration membranes three times, and dialyzed for 1 day in dialysis bags (MWCO 1,000 Da). The obtained brownish-black solutions were evaporated and lyophilized to get brown solids. The solids were dissolved in phosphate buffer (pH = 7.40, 0.01 M) to obtain Se/N-CQDs (1 mg/mL) for further applying.

### Study on the Scavenging Ability of Se/N-CQDs to ABTS^•+^

**Scheme 2:**
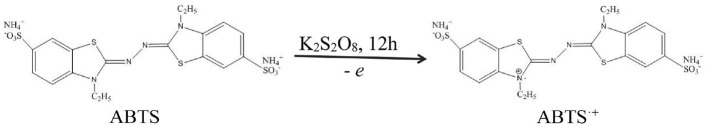
Schematic of the preparation of ABTS^•+^.

ABTS^•+^ as the abbreviation of the free radical of ABTS ([2,2'-azinobis (3-ethylbenzothiazoline-6-sulphonic acid ammonium salt)]) was generated by ABTS and K_2_S_2_O_8_ in the dark (Scheme 2). In total, 3.4 mg of potassium persulfate and 20 mg of ABTS were dissolved in 5.21 mL phosphate buffer (pH = 7.40, 0.01 M), and the mixed solutions were allowed to stay for 12 h under dark conditions to prepare ABTS^•+^ aqueous solutions (Jibril et al., 2017). This is because the concentration of ABTS^•+^ reached a maximum after 6 h and was stable for more than 2 days when stored in the dark at room temperature (Re, 1999; Kusznierewicz et al., 2012). The solutions were diluted with phosphate buffer (pH = 7.40, 0.01 M). The UV-Vis absorbance of ABTS^•+^ was measured as 0.70 ± 0.02 at 734 nm by a UV-3600 UV-VIS-NIR spectrophotometer.

Trolox (6-hydroxy-2,5,7,8-tetramethylchro-man-2-carboxylic acid) is a water-soluble a-tocopherol derivative often used as a control to compare the antioxidant capacity of other compounds (Poljsak et al., 2006; Alberto et al., 2013; Kitasaka et al., 2020). Trolox was selected as the control sample. Different concentrations of Se/N-CQDs and Trolox (0.01, 0.02, 0.025, 0.033, 0.05, 0.10, 0.20, 0.30, 0.40, 0.50, 0.60, 0.70, 0.80, 0.90, and 1.00 mg/mL) were diluted by phosphate buffer (pH = 7.40, 0.01 M) and added to ABTS^•+^ solutions. The absorbance at 734 nm was recorded under the same conditions in a few minutes. If the absorbance A_734 nm_ decreased, ABTS^•+^ was scavenged (Re, 1999). The percentage radical scavenging activity of Se/N-CQDs was calculated using the following formula:

(1) $$\begin{array}{l} \text{Scavenging activity }\left(\%\right)=\left(\text{A}0_{734}-{\text{A}}_{734}\right)/{\text{A}0}_{734}*100\% \end{array}$$

where “*A0*_*734*_” was the absorbance of ABTS^•+^ without the addition of Se/N-CQDs or Trolox. “*A*_*734*_” was the absorbance of ABTS^•+^ with the addition of Se/N-CQDs or Trolox.

### Effect of Metal Cations on the Ability of Se/N-CQDs to Scavenge ABTS^•+^

To investigate the effect of metal cations on the ability of Se/N-CQDs to scavenge ABTS^•+^, 250 μL ultra-pure water and various metal cations, Ca(II), Co(II), Cr(VI), Cr(III), Cu(II), Fe(II), K(I), Mn(II), Mg(II), Na(I), Ni(II), Pb(II) and Zn(II), and 1 mM, were added into the 800 μL Se/N-CQDs + ABTS^•+^ mixed solutions (Mixed solutions) at 734 nm under the same conditions. Se/N-CQDs + ABTS^•+^ mixed solutions (Mixed solutions) consisted of 40 mL ABTS^•+^ (A_734 nm_ = 0.70) and 500 μL Se/N-CQDs (1 mg/mL). The UV-Vis absorption spectra were recorded by a UV-3600 UV-VIS-NIR spectrophotometer under the same conditions.

The effect of metal cations on the absorbance of Se/N-CQDs or ABTS^•+^ was also studied. In total, 1 mL ultra-pure water and various metal cations, Ca(II), Co(II), Cr(VI), Cr(III), Cu(II), Fe(II), K(I), Mn(II), Mg(II), Na(I), Ni(II), Pb(II) and Zn(II), and 1 mM, were added into the 200 μL Se/N-CQDs solutions (1 mg/mL). In total, 250 μL ltra-pure water and various metal cations, Ca(II), Co(II), Cr(VI), Cr(III), Cu(II), Fe(II), K(I), Mn(II), Mg(II), Na(I), Ni(II), Pb(II) and Zn(II), and 1 mM, were added into the 800 μL ABTS^•+^ solutions. The UV-Vis absorption spectra were recorded by a UV-3600 UV-VIS-NIR spectrophotometer under the same conditions.

### Sensitivity of Se/N-CQDs and ABTS^•+^ Mixed Solutions for Mn(II) Detection

The sensitivity of Se/N-CQDs and ABTS^•+^ solutions for Mn(II) detection was determined by adding 100 μL Mn(II) (1 mM) solutions into 3 mL Se/N-CQDs + ABTS^•+^ mixed solutions (Mixed solutions) orderly at 734 nm. The concentrations of Mn(II) were 0, 32.20, 62.50, 90.90, 117.60, 142.90, 166.70, and 189.20 μM. The UV-Vis absorption spectra were recorded by a UV-3600 UV-VIS-NIR spectrophotometer under the same conditions.

### Synergy of Mn(II) and Se/N-CQDs

The synergy of Mn(II) and Se/N-CQDs was measured by adding Mn(II) (1 mM) and Se/N-CQDs (1 mg/mL) into ABTS^•+^, and four groups of the experimental data are shown in Supplementary Table 1. The UV-Vis absorption spectra were recorded by a UV-3600 UV-VIS-NIR spectrophotometer under the same conditions. All solutions were prepared in ultra-pure water at room temperature.

### Detection of Mn(II) in Real Sample

To evaluate the feasibility of Se/N-CQDs and ABTS^•+^ used as a colorimetric probe for Mn(II) detection in a real sample, tap water obtained from our lab with no further processing was selected as the real sample. Mn(II) tap solutions were prepared by adding MnCl_2_·4H_2_O into tap water at room temperature. The sensitivity of Se/N-CQDs and ABTS^•+^ solutions for Mn(II) detection was confirmed by adding 50 μL Mn(II) (1 mM) solutions into 3 mL Se/N-CQDs + ABTS^•+^ mixed solutions (Mixed solutions) orderly at 734 nm. The concentrations of Mn(II) were 0, 16.40, 32.20, 47.60, 62.50, 76.90, 90.90, 104.50, 117.60, and 130.40 μM. The UV-Vis absorption spectra were recorded by a UV-3600 UV-VIS-NIR spectrophotometer under the same conditions.

## Results and Discussion

### Characterizations of Se/N-CQDs

**Figure 1:**
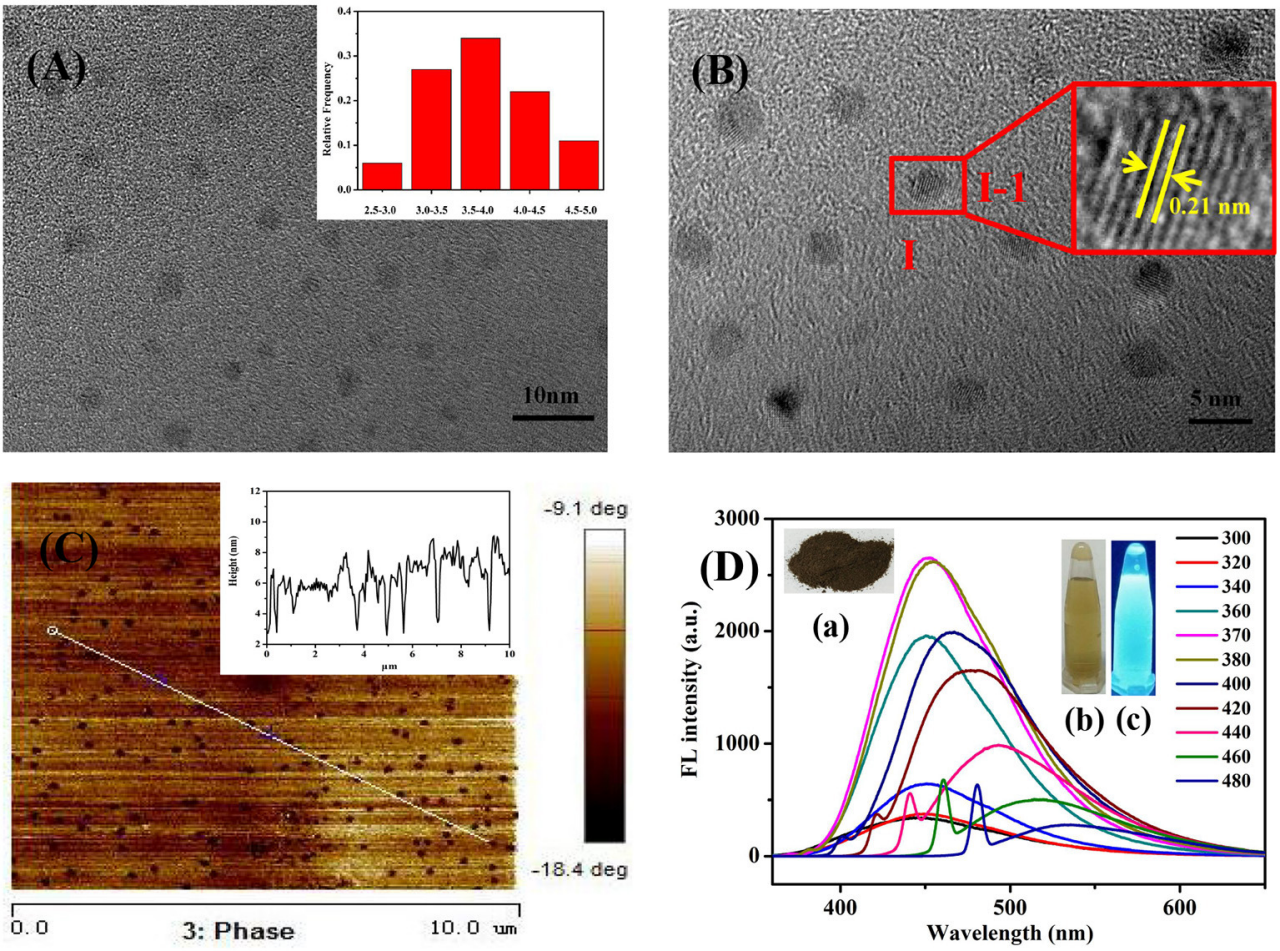
**(A)** TEM image of Se/N-CQDs. Insert: Diameter distribution histogram of Se/N-CQDs. **(B)** HRTEM image of Se/N-CQDs. Insert: (I-1) Magnified image of “I” Se-CQD. **(C)** AFM image of Se/N-CQDs. Insert: AFM height profile analysis along the corresponding line in **(C)**. **(D)** Emission spectra of Se/N-CQDs at different excitation wavelengths. Inserts: (a) The powder photograph of Se/N-CQDs; (b) the photographs of Se/N-CQDs solutions under the white light irradiation; (c) the photographs of the preparation of Se/N-CQDs solutions under the UV (365 nm) light irradiation.

The TEM and AFM characterizations of Se/N-CQDs were shown in Figures 1A–C, revealed that Se/N-CQDs were spherical, dispersive, and stable. From the particle diameter distribution histogram in Figure 1A insert and the AFM height profile analysis along the corresponding line in Figure 1C, the average diameter of Se/N-CQDs was 4 ± 1 nm, which was similar to the value reported in the literature (Zhang and Chen, 2014; Jiang et al., 2015; Sharma et al., 2016). One Se-CQD in Figure 1B insert (I-1) showed a crystalline structure with a lattice spacing of 0.21 nm, which was attributed to the (110) diffraction planes of graphitic carbon (JCPDS cards 26-1076) (Yang et al., 2014). The High-resolution XPS spectrum of C_1s_ was shown in Supplementary Figure 1A, the deconvoluted peaks located at 284.6, 285.1, and 287.7 eV were attributed to C-C/C=C, C-N/C-H, and O-C=O groups, respectively (Zhang and Chen, 2014; Zhou et al., 2015; Shen et al., 2017). As shown in Supplementary Figure 1B, the high-resolution XPS spectrum of N_1s_ spectrum could be deconvoluted into N-C (398.5 and 400.0 eV) functional groups (Zhang and Chen, 2014; Bankoti et al., 2017; Shen et al., 2017). As seen in Supplementary Figure 1C, there were two deconvoluted peaks at 530.8 and 531.9 eV, representing the presence of C-O and C=O/O-H bonds, respectively, in the high-resolution XPS spectrum of O_1s_ spectrum (Zhang et al., 2015; Bankoti et al., 2017). It can be seen from Supplementary Figure 1D that the deconvoluted peak at 1,071.3 eV in the Na_1s_ XPS spectrum was attributed to Na^+^ (Guo et al., 2017). The weak deconvoluted peak located at 53.2 eV was attributed to Se^2−^ in the high-resolution XPS spectrum of Se_3d_ spectrum in Supplementary Figure 1E (Ray et al., 2013), illustrating the existence of selenium elements in Se/N-CQDs. As shown in Supplementary Figure 1F, the result of EDS displayed that Se/N-CQDs were composed of C, O, N, Na, and Se elements, which were in good agreement with the XPS characterization results. Supplementary Figure 2A was the ^1^H NMR spectrum of Se/N-CQDs. Signals between 6 and 9 ppm belong to sp^2^ hybridized carbon atoms, illustrating the aromatic structures of Se/N-CQDs (Li et al., 2017). ^13^C NMR spectrum of Se/N-CQDs was shown in Supplementary Figure 2B, signals between 25 and 70 ppm were attributable to aliphatic (sp^3^) carbon atoms. Signals between 100 and 180 ppm were ascribed to sp^2^ hybridized carbon atoms (Bankoti et al., 2017; Li et al., 2017). Especially, signals between 160 and 180 ppm should be put down to carboxy/amide groups on the surface of Se/N-CQDs (Bankoti et al., 2017), which was in line with the results of XPS and FTIR (Supplementary Figure 4). In addition, the hybridization of the carbon atoms was further characterized by Raman. It could be seen from Supplementary Figure 2C, there were two peaks at 1,342 and 1,573 cm^−1^, which corresponded to the D band (sp^3^) and G band (sp^2^), respectively (Chunduri et al., 2016). The D band was associated with vibrations of carbon atoms with a dangling bond in the termination plane of disordered graphite. The G band was attributed to the ordered graphite structure (Chunduri et al., 2016; Bankoti et al., 2017). The ratio of I_*D*_/I_*G*_ was 1.15, which was the characteristic of the disorder and the ratio of sp^3^/sp^2^ carbon atoms, implying that structural defects were introduced into Se/N-CQDs (Chunduri et al., 2016). The results of Raman and NMR collectively indicated that there were sp^3^/sp^2^ carbon atoms in Se/N-CQDs. As shown in Figure 1D, the brown Se/N-CQDs solids [insert (a) were dissolved quickly in phosphate buffer (pH = 7.40, 0.01 M), showing the good water solubility (insert (b)] and the bright blue fluorescence under UV light irradiation [insert (c), 365 nm]. As shown in Figure 1D, the fluorescence peaks shifted from 435 to 535 nm with the increase of excitation wavelength from 300 to 480 nm, illustrating that Se/N-CQDs exhibited an excitation-dependent fluorescence behavior, which was attributed to the optical selection of differently sized nanoparticles and different emissive traps on the surface of CQDs or another mechanism (Shang et al., 2012; Madrakian et al., 2017). The maximum fluorescence intensity of Se/N-CQDs was at the emission wavelength of 450 nm when the excitation wavelength was about 370 nm. By selecting quinine sulfate as a criterion, the fluorescence quantum yield of Se/N-CQDs was 6.43%, which was close to the value reported in the literature (Shi et al., 2014; Meng et al., 2018).

### Study on the Antioxidant Capacity of Se/N-CQDs

**Figure 2:**
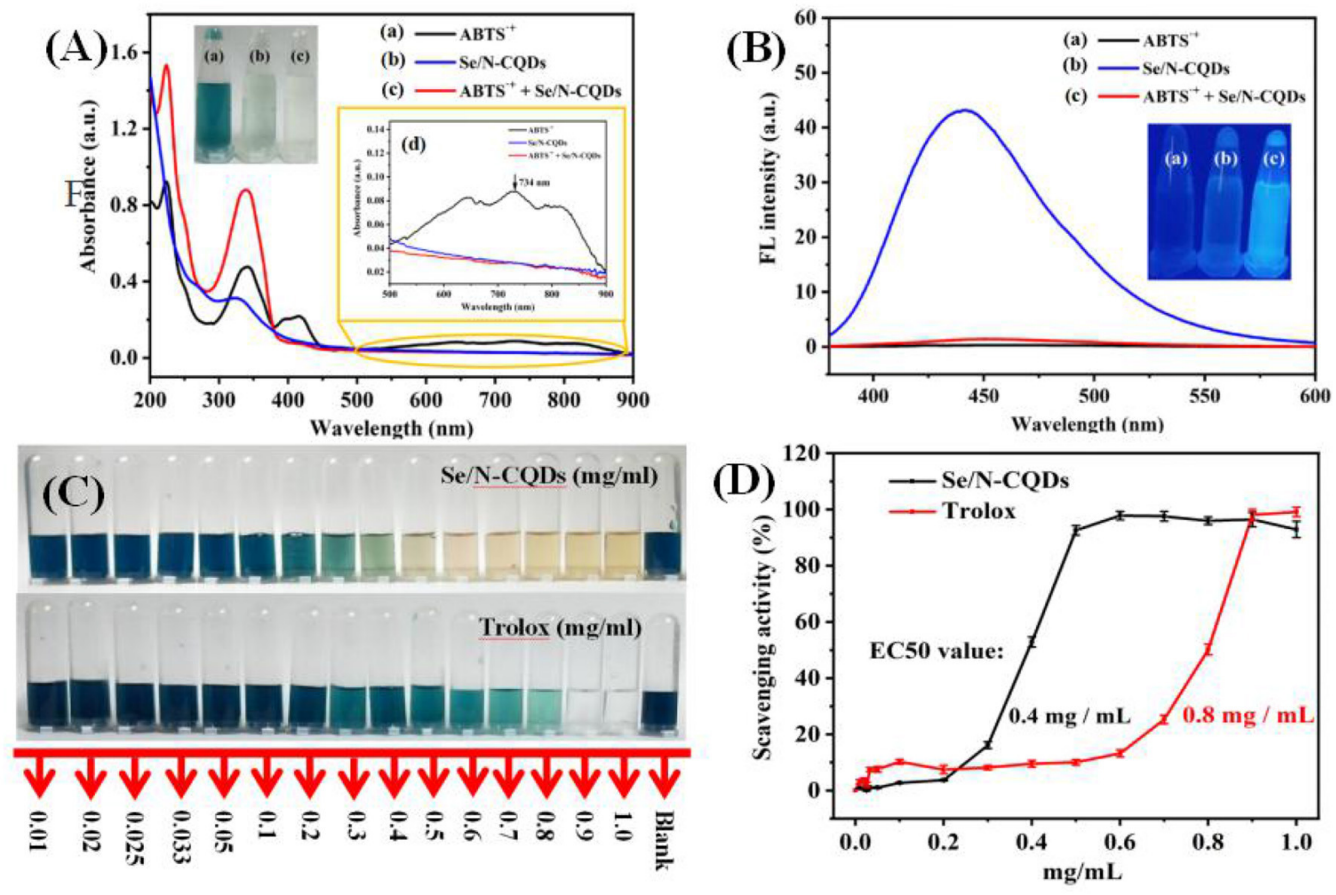
**(A)** UV-Vis absorption spectra of ABTS^•+^, Se/N-CQDs and ABTS^•+^ + Se/N-CQDs. Inserts: The photographs of ABTS^•+^ (a), Se/N-CQDs (b), and ABTS^•+^ + Se/N-CQDs (c) solutions under the white light irradiation, and (d) partial enlargement of UV-vis absorption spectra. **(B)** Emission spectra of ABTS^•+^, Se/N-CQDs, and ABTS^•+^ + Se/N-CQDs. Inserts: the photographs of ABTS^•+^ (a), Se/N-CQDs (b), and ABTS^•+^ + Se/N-CQDs (c) solutions under the UV (365 nm) light irradiation. **(C)** Photographic representations of bleaching of ABTS^•+^ solutions with a progressive increase in the concentration of Se/N-CQDs and Trolox, respectively. **(D)** ABTS^•+^ free radical scavenging activities of Se/N-CQDs and Trolox, respectively.

As shown in Figure 2A and inserts, when Se/N-CQDs were added into ABTS^•+^, the peak at 734 nm decreased or disappeared with the fading of blue. In Figure 2B, Se/N-CQDs exhibited the blue fluorescence at λ ex = 370 nm, while ABTS^•+^ could not emit fluorescence. When Se/N-CQDs were added into ABTS^•+^, the fluorescence of Se/N-CQDs was quenched. It illustrated that Se/N-CQDs and ABTS^•+^ could directly interact with each other, leading to the fluorescence quenching and changes in UV-Vis absorption spectra. The reason for Se/N-CQDs scavenging ABTS^•+^ was further studied. As long as the redox potential of the compound was lower than 0.68 V, which was the redox potential of ABTS^•+^ (Vera et al., 2017), ABTS^•+^ could be reduced, resulting in the blue degradation of the system. The reduction peak of Se/N-CQDs in phosphate buffer (pH = 7.20) was located at 0.27 V in Supplementary Figure 3. As a result, the redox reaction could occur between Se/N-CQDs and ABTS^•+^ with the fading of blue. In addition, the Zeta potential of Se/N-CQDs was −13.8 mV in phosphate buffer (pH = 7.4, 0.01 M). A negative charge on the surface of Se/N-CQDs was beneficial to the reaction between ABTS^•+^ and Se/N-CQDs. It could be seen from Figure 2C, the radical scavenging activities of Se/N-CQDs and Trolox were found to increase in a dose-dependent manner. The ABTS^•+^ solutions turned blue to yellow when the concentration of Se/N-CQDs was about 0.5 mg/mL. While ABTS^•+^ solutions turned blue to transparent when the concentration of Trolox was about 0.9 mg/mL. As shown in Figure 2D, with the concentration of Se/N-CQDs and Trolox increased from 0.2 to 0.5 mg/mL, there was a subsequent increase in the scavenging activity from 3 to 93% for Se/N-CQDs. However, there was almost no obvious change in the scavenging activity from 7 to 10% for Trolox under the same conditions. The EC50 value (amount of antioxidant required to decrease the concentration of ABTS^•+^ by 50%) of Se/N-CQDs and Trolox were estimated to be 0.4 and 0.8 mg/mL from the curve, respectively. It showed that the radical scavenging activity of Se/N-CQDs was greater than that of Trolox, exhibiting admirable antioxidation capacity of Se/N-CQDs.

### Effect of Metal Cations on the Ability of Se/N-CQDs to Scavenge ABTS^•+^

**Figure 3:**
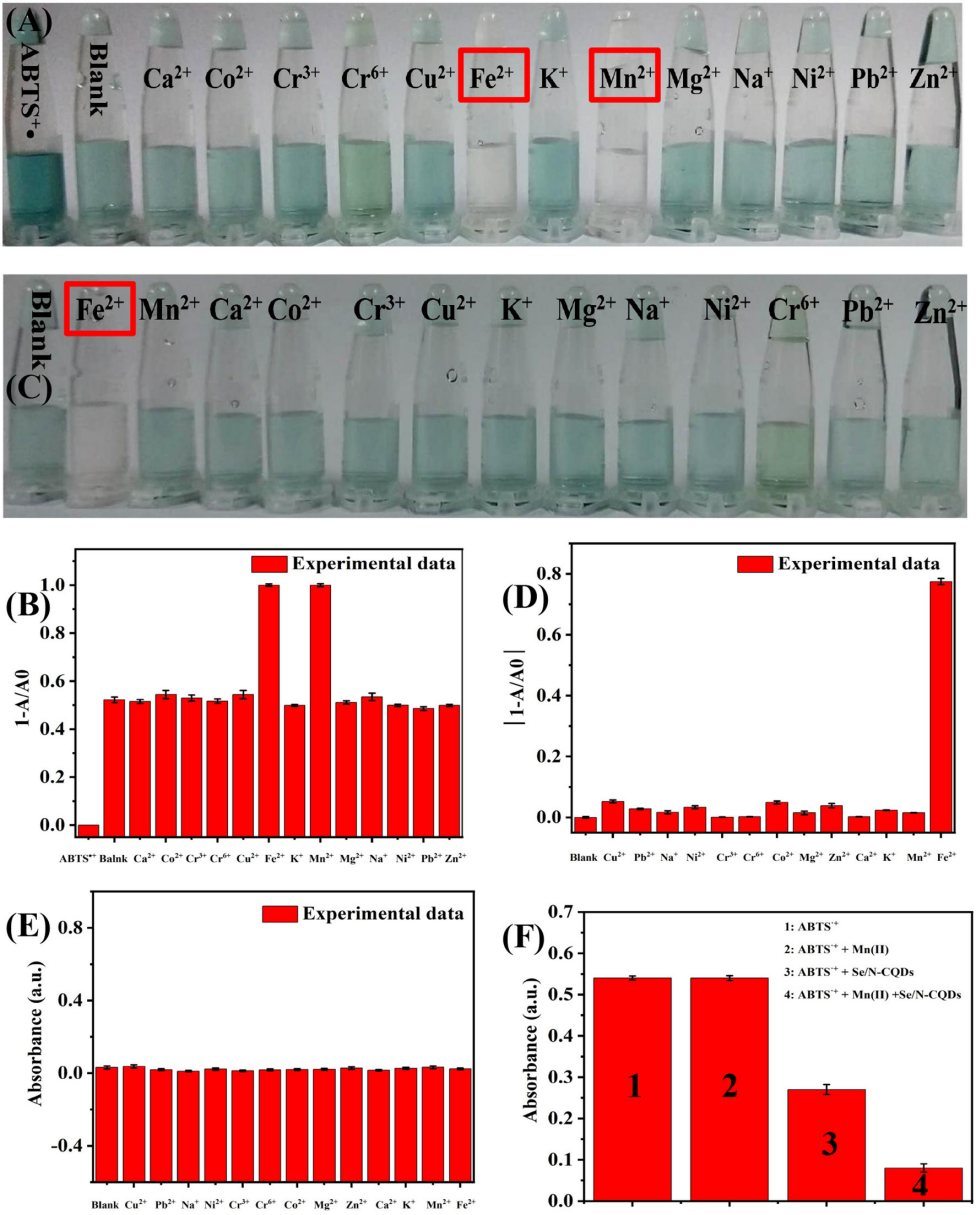
**(A)** Photographs of ABTS^•+^ and Se/N-CQDs + ABTS^•+^ mixed solutions (Mixed solutions) containing various metal cations when a small amount of Se/N-CQD was added into ABTS^•+^. **(B)** The relationship between 1-A/A0 and various metal cations at 734 nm. A was the UV-Vis absorbance of Mixed solutions containing water and various metal cations. A0 was the UV-Vis absorbance of ABTS^•+^. **(C)** Photographs of ABTS^•+^ containing various metal cations. **(D)** The relationship between 1-A/A0 and various metal cations at 734 nm. A was the UV-Vis absorbance of ABTS^•+^ solutions containing various metal cations. A0 was the UV-Vis absorbance of ABTS^•+^ solutions containing water. **(E)** The UV-Vis absorbance of Se/N-CQDs containing water and various metal cations at 734 nm. **(F)** The UV-Vis absorbance of ABTS^•+^, ABTS^•+^ + Mn(II), ABTS^•+^ + Se/N-CQDs, and ABTS^•+^ + Mn(II) + Se/N-CQDs.

It could be seen from Figures 3A,B that when a small amount of Se/N-CQD was added to ABTS^•+^, ABTS^•+^ could be only scavenged in the presence of Fe(II) and Mn(II), while other metal cations, Ca(II), Co(II), Cr(VI), Cr(III), Cu(II), Fe(II), K(I), Mn(II), Mg(II), Na(I), Ni(II), Pb(II), and Zn(II), had no effect on the ability of Se/N-CQDs to scavenge ABTS^•+^. What's more, as shown in Figures 3C,D, when various metal cations were added into ABTS^•+^, ABTS^•+^ could be only scavenged in the presence of Fe(II) at 734 nm, while ABTS^•+^ could not be scavenged in the presence of Mn(II) at 734 nm. Moreover, when various metal cations were added, Se/N-CQDs had almost no absorbance, indicating that Se/N-CQDs might not affect the absorbance of ABTS^•+^ at 734 nm in Figure 3E. Therefore, Fe(II) and Mn(II) could be distinguished by Se/N-CQDs and ABTS^•+^. It also illustrated that ABTS^•+^ and Se/N-CQDs as the colorimetric probe could be applied to detecting Mn(II) ions. As shown in Figure 3F, the change of the absorbance of ABTS^•+^ containing Mn(II) and Se/N-CQDs was greater than the sum of the changes of the absorbance of ABTS^•+^ containing Mn(II) and the absorbance of ABTS^•+^ containing Se/N-CQDs at 734 nm, indicating that Mn(II) and Se/N-CQDs had a synergistic effect on the removal of ABTS^•+^.

### Sensitivity of Se/N-CQDs and ABTS^•+^ Mixed Solutions for Mn(II) Detection

**Figure 4:**
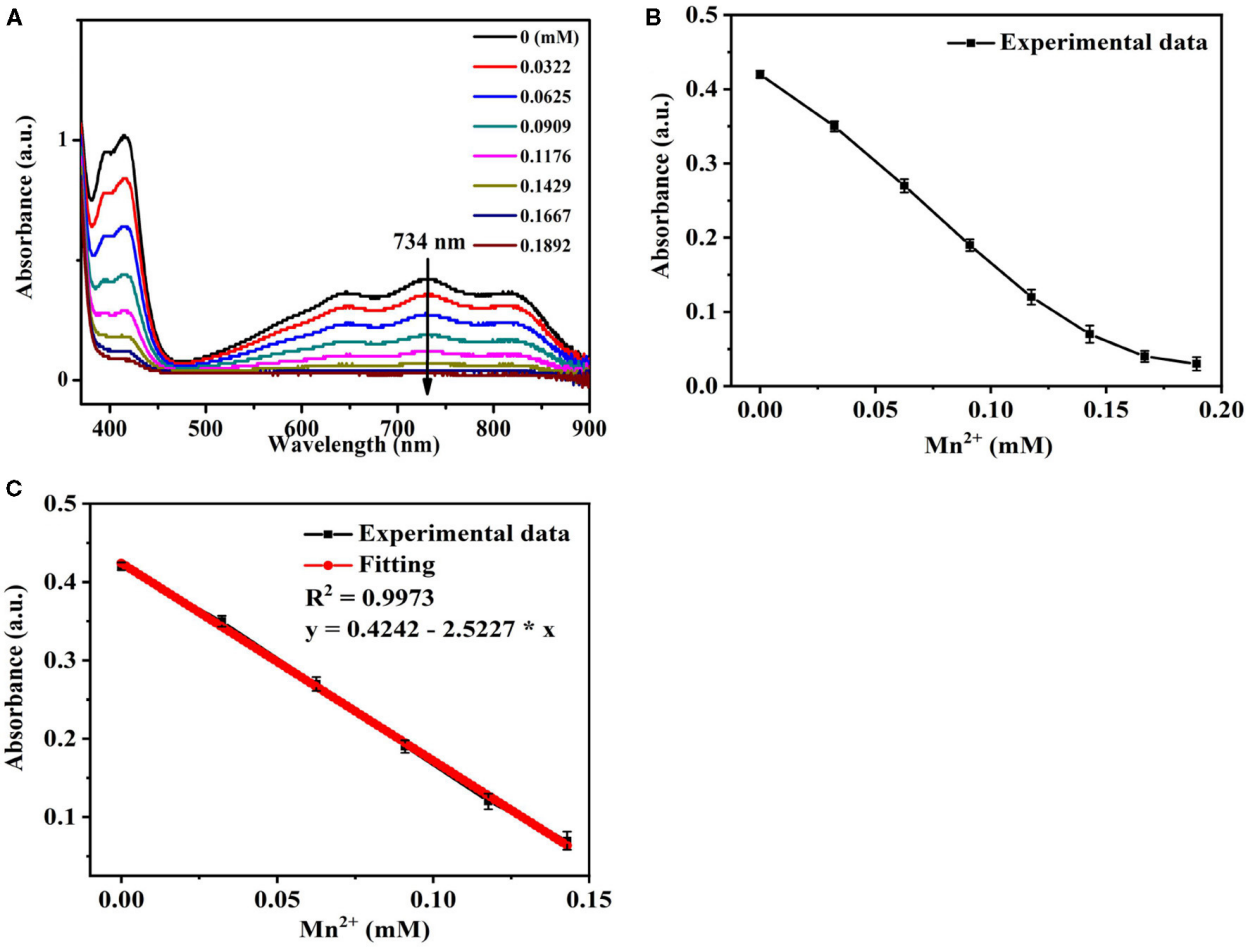
**(A)** The UV-Vis absorption spectra of Se/N-CQDs + ABTS^•+^ mixed solutions (Mixed solutions) in the presence of different concentrations of Mn(II). **(B)** The UV-Vis absorbance of Mixed solutions in the presence of different concentrations of Mn(II) in the range of 0 to 189.20 μM at 734 nm. **(C)** The linear relationship between the UV-Vis absorbance of Mixed solutions and the concentrations of Mn(II) in the range of 0 to 142.90 μM at 734 nm.

It could be seen from Figures 4A,B, the UV-Vis absorbance of Se/N-CQDs + ABTS^•+^ mixed solutions (Mixed solutions) decreased with the increase of Mn (II) at 734 nm. The linear regression equation with a correlation coefficient of 0.9973 showed good linearity in the range of 0–142.90 μM in Figure 4C. The limit of detection (LOD = 3 Sk/k, Sk as the standard deviation and k as the slope) was calculated to be 1.69 μM: this was lower than those previously reported values in Supplementary Table 2 and less than the corresponding national drinking water standard (1.80 μM) and the WHO guideline of drinking water (7.28 μM) (Lee et al., 2014). The different concentrations of Mn(II) (62.50, 90.90, 117.60, and 142.90 μM) were added into Se/N-CQDs + ABTS^•+^ mixed solutions 1, and their maximum absorbances were acquired. Each sample was measured three times, and the detection results were average values. The analytical results of Mn(II) determined by the probe based on the absorbance of Mixed solutions were 66.00, 95.90, 122.00, and 138.00 μM, which were consistent with the added Mn(II) concentrations in Supplementary Table 3. It illustrated that ABTS^•+^ and Se/N-CQDs colorimetric probe had excellent sensitivity, selectivity, stability, and reproducibility for the detection of Mn(II).

### Sensitivity of Se/N-CQDs and ABTS^•+^ Mixed Solutions for Mn(II) Detection in Tap Water

The feasibility of ABTS^•+^ and Se/N-CQDs for the detection of Mn(II) in real samples was further evaluated, and tap water obtained from our lab with no further processing was selected as a real sample. Supplementary Figures 4A,B show the absorbance of Se/N-CQDs + ABTS^•+^ mixed solutions (Mixed solutions) also decreased with the increase of Mn (II) in tap water at 734 nm. There was a good linear calibration with a correlation coefficient of 0.9996 under the Mn(II) ion concentration range from 0 to 104.50 μM at 734 nm in Supplementary Figure 4C. The limit of detection (LOD) was calculated to be 1.24 μM, which was also less than the corresponding national drinking water standard (1.80 μM) and the WHO guideline of drinking water (7.28 μM). When the different concentrations of Mn(II) (32.20, 47.60, 62.50, and 76.90 μM) in tap water were added into Mixed solutions, the tap water samples with Mn(II) additions were measured by using the colorimetric sensor based on ABTS^•+^ + Se/N-CQDs and Inductively Coupled Plasma Optical Emission Spectrometry (ICP-OES, Agilent, ICP/700) as the standard method. The analytical results of Mn(II) determined by the colorimetric sensor based on ABTS^•+^ + Se/N-CQDs and ICP-OES are listed in Supplementary Table 4. Each sample was measured three times, and the detection results were average values. It can be seen from Supplementary Table 4 that the analytical results of Mn(II) determined by the colorimetric sensor based on ABTS^•+^ + Se/N-CQDs were consistent with the analytical results of Mn(II) determined by ICP-OES methods, indicating that the colorimetric sensor based on ABTS^•+^ and Se/N-CQDs solutions was accurate and had the application potential for the Mn(II) detection in real sample.

## Conclusion

In this work, a one-step method was applied to synthesize Se/N-CQDs by using citric acid and histidine as carbon sources and sodium selenite as a selenium source. The as-prepared Se/N-CQDs not only had sp^3^/sp^2^-hybridized carbon atoms but also exhibited the excitation-dependent fluorescence behavior with the maximum excitation and emission at 370 and 450 nm, respectively. Moreover, Se/N-CQDs exhibited admirable antioxidation capacity by scavenging ABTS^•+^. Due to the synergistic effect of Se/N-CQDs and Mn(II) on ABTS^•+^, Se/N-CQDs and ABTS^•+^ as an excellent colorimetric sensor for determination of Mn(II) showed a linear relationship with LOD of 1.69 μM from 0 to 142.90 μM. The probe was also successfully applied to the detection of Mn(II) in tap water. It is hoped that Se/N-CQDs and ABTS^•+^ could be a promising sensing platform for the colorimetric detection of Mn(II) with a wide range of potential applications. It is also expected that not only the fluorescence properties of carbon quantum dots but also other properties, such as antioxidant properties, can be exploited to increase the application ability of carbon quantum dots.

## Data Availability Statement

The original contributions presented in the study are included in the article/Supplementary Material, further inquiries can be directed to the corresponding author/s.

## Author Contributions

QX: data curation and writing—original draft preparation. XL and YJ: writing. PW: writing—reviewing and editing. All authors contributed to the article and approved the submitted version.

## Conflict of Interest

The authors declare that the research was conducted in the absence of any commercial or financial relationships that could be construed as a potential conflict of interest.
