# Supplementary material for: A Highly Sensitive and Selective Probe for the Colorimetric Detection of Mn(II) Based on the Antioxidative Selenium and Nitrogen Co-Doped Carbon Quantum Dots and ABTS•+
Source: Front Chem. 2021 Jul 2;9:658105. doi: 10.3389/fchem.2021.658105 (PMC8282897; doi:10.3389/fchem.2021.658105)
Supplement: Supplementary file 1 [file Data_Sheet_1.pdf]

Supplementary Material

A Highly Sensitive and Selective Probe for the Colorimetric Detection of Mn(II) Based on the Antioxidative Selenium and Nitrogen Co-doped Carbon Quantum Dots and ABTS$^{•+}$

Qinhai Xu [a], Xiaolin Liu [a], Yanglin Jiang [a], Peng Wang [a]*

[a] Department of Chemistry, Renmin university of China, Beijing 100872, china

* Corresponding author, E-mail address: wpeng_chem@ruc.edu.cn

Tel.: +86 10 62516604.

S1. Experimental

S1.1. Materials and reagents

Citric acid ($C_6H_8O_7$), L-Histidine ($C_6H_9N_3O_2$), Cobalt chloride hexahydrate ($CoCl_2·6H_2O$), Iron sulfate heptahydrate ($FeSO_4·7H_2O$), Manganese chloride ($MnCl_2·4H_2O$), Nickel sulfate hexahydrate ($NiSO_4·6H_2O$), ABTS [2,2'-azinobis (3-ethylbenzothiazoline-6-sulphonic acid ammonium salt)], Dimethyl sulfoxide (C_2H_6SO) were purchased from Aladdin Industrial Corporation, (Shanghai, China). Sodium selenite anhydrous (Na_2SeO_3) was bought from Alfa Aesar (China) Chemical Co., Ltd., (Shanghai, China). Chromic chloride hexahydrate ($CrCl_3·6H_2O$) was purchased from Adamas Reagent Co., Ltd., (Shanghai, China). Quinine sulfate dihydrate was bought from Shanghai Titan Scientific Co., Ltd., (Shanghai, China). Trolox (6-hydroxy-2,5,7,8-tetramethylchro-man-2-carboxylic acid) was obtained from Sigma-Aldrich Co., Ltd., (Russian Fed). Calcium chloride anhydrous ($CaCl_2$), Magnesium chloride hexahydrate ($MgCl_2·6H_2O$), Sodium chloride (NaCl) were purchased from Sinopharm Chemical Reagent Co., Ltd., (Beijing, China). $Na_2HPO_4·12H_2O$, $NaH_2PO_4·2H_2O$, Potassium peroxydisulfate ($K_2S_2O_8$), Potassium dichromate ($K_2Cr_2O_7$), Potassium chloride (KCl), Sodium hydroxide (NaOH) and Zinc Chloride ($ZnCl_2$) were bought from Beijing Chemical Works (Beijing, China). Cupric sulfate, anhydrous ($CuSO_4$) was obtained from Beijing Yili Chemical Co., Ltd., (Beijing, China). Lead Chloride ($PbCl_2$) was purchased from Tianjin KERMEL Chemical Reagents Development Centre (Tianjin, China). The Ca(II), Co(II), Cr(VI), Cr(III), Cu(II), Fe(II), K(I), Mn(II), Mg(II), Na(I), Ni(II), Pb(II) and Zn(II)

solutions (1mM) were prepared in ultra-pure water from the salts of $CaCl_2$, $CoCl_2 \cdot 6H_2O$, $K_2Cr_2O_7$, $CrCl_3 \cdot 6H_2O$, $CuSO_4$, $FeSO_4 \cdot 7H_2O$, KCl, $MnCl_2 \cdot 4H_2O$, $MgCl_2 \cdot 6H_2O$, NaCl, $NiSO_4 \cdot 6H_2O$, $PbCl_2$ and $ZnCl_2$, respectively. All chemicals were analytical grade and all solutions were prepared by ultra-pure water.

S1.2. Apparatus

Transmission electron microscopy (TEM) and high-resolution TEM (HRTEM) were conducted with operaing voltage of 300 kV on a TECNAI transmission electron microscope. Atomic force microscope (AFM) images were collected by using a Bruker Dimension Icon AFM equipped with Scanasyst in Air peak force tapping mode AFM tips from Bruker. Raman spectroscopy measurement was carried out using Raman spectroscopy with laser excitation of 532 nm at room temperature (HORIBA Scientific, Xplora plus, France). X-ray photoelectron spectroscopy (XPS) were obtained by a Thermo ESCALAB 250Xi spectrometer (Setting parameters: monochromatic Al Ka (hv = 1486.6 eV), operated at 150 W, 500 μm beam spot, the binding energy was calibrated against the carbon 1s line at 284.8 eV, USA). Fourier-transformed infrared spectroscopy (FTIR) was taken on a IRPrestige-21 (SHIMADZU, KBr wafer technique, Japan). Fluorescence spectra were recorded on a fluorescence Spectrophotometer (HITACHI F-4600, Japan). The lifetime of fluorescence was obtained by an Edinburgh FLS980 all functional steady-state/transient fluorescence spectrometer (Edinburgh Instruments, UK). UV-Vis spectrum was obtained a UV-3600 UV-VIS-NIR spectrophotometer (SHIMADZU, Japan). Zeta potential was measured by using the Nano-ZS90

zetasizer (Malvern Instruments Corp, UK.). Thermogravimetric (TG) analysis was performed on a TA company TGA Q500 unit in N_2 at a heating rate of 10 °C min^{-1} from room temperature to 800 °C. XRD spectrum was measured by using the XRD-7000 X-RAY DIFFRACTOMETER (SHIMADZU, Japan). ^{1}H NMR and ^{13}C NMR spectra were recorded on the Advance Bruker 400M NMR spectrometer. Ultra-pure water was obtained from a Millipore Mingche™-D 24^{UV} water purification system.

The electrochemical measurement was measured by using a AUTOLAB electrochemical workstation (Autolab PGSTAT302N, Metrohm, Switzerland) in a typical three electrode, a graphite rod as the counter electrode, a glassy carbon electrode coated with Se/N-CQDs as the working electrode and a saturated calomel electrode (SCE) as the reference electrode. The electrode was polished with polishing powder, washed with deionized water, and then dried with cold air. Then, 4 mg Se/N-CQDs powder, 4 mg carbon black were admixed in 750 μL deionized water, 250 μL ethanol, 30 μL 5 wt% Nafion solution, forming a mixed solution, which was ultrasound for at least 30 min, 5 μL the prepared solution was dropped onto the polished electrode eventually. The redox properties of Se/N-CQDs were investigated in phosphate buffer (pH = 7.20） with the loading of 0.5 mg cm^{-2} on a glassy carbon (GC) electrode. CV curve was performed at a potential range of -0.5 – 1.5 V vs RHE and at a scan rate of 100 mV/s.

S1.3. The measurement of quantum yield (QY) of Se/N-CQDs

The reference method was used to determine the quantum yield of Se/N-CQDs [S1]. Quinine sulfate in 0.1 M H_2SO_4 solution (quantum yield of 53.0% excited at 365 nm) was selected as a criterion by the following equation:

$$\Phi = \Phi_s [(I / I_s) (A_s / A) (n^2 / n_s^2)] \qquad (1)$$

Where "Φ" is the quantum yield, "I" is the fluorescence peak area at the maximum excitation wavelength, "A" is the UV-Vis absorbance intensity，the subscript "S" represents the reference quinine sulfate, "n" is the different refractive index of solvent, the superscript "2" means the square of "n".

S1.4. Study on the fluorescence intensity of Se/N-CQDs

3 mL of Se/N-CQDs (1 mg/mL) were continuously measured for 20 counts to investigate the stability of the fluorescence intensity of Se/N-CQDs under the same conditions. In order to study the effect of concentration on the fluorescence intensity of Se/N-CQDs, the prepared Se/N-CQDs (1 mg/mL) were diluted to 0.9, 0.8, 0.7, 0.6, 0.5, 0.4, 0.3, 0.2, 0.1, 0.05 times with ultra-pure water. The effect of pH on the fluorescence intensity of Se/N-CQDs was further discussed. The aqueous solutions at different pH (2.60, 3.46, 4.23, 5.10, 6.03, 7.25, 8.36, 9.36, 10.35, 11.16, 12.03) were prepared by using sodium hydroxide and concentrated sulfuric acid. 3 mL of the aqueous solutions at different pH and 3 mL of ultra-pure water were added into 1 mL of Se/N-CQDs, respectively. The fluorescence intensities were recorded on the fluorescence spectrophotometer (HITACHI F-4600) at the excitation

wavelength of 370 nm. All solutions were prepared in ultra-pure water at room temperature.

S1.5. Effect of ionic strength on the ability of Se/N-CQDs to scavenge $ABTS^{•+}$

To discuss the effect of ionic strength on the ability of Se/N-CQDs to scavenge $ABTS^{•+}$, NaCl with the different orders of magnitude and the same order of magnitude were added into the $ABTS^{•+}$ + Se/N-CQDs mixed solutions. 250 μL ultra-pure water and different concentrations of NaCl with the different orders of magnitude (0.000000001, 0.00000001, 0.0000001, 0.000001, 0.00001, 0.0001, 0.001, 0.01, 0.1, 1 M) were added into the 800 μL $ABTS^{•+}$ + Se/N-CQDs mixed solutions S1 at 734 nm under the same conditions, respectively, and Se/N-CQDs + $ABTS^{•+}$ mixed solutions S1 were composed of 40 mL $ABTS^{•+}$ ($A_{734\ nm}$ = 0.70) and 500 μL Se/N-CQDs (1 mg/mL). In addition, 250 μL ultra-pure water and different concentrations of NaCl with the same order of magnitude (0, 0.1, 0.2, 0.3, 0.4, 0.5, 0.6, 0.7, 0.8, 0.9, 1.0 mM) were added into the 800 μL $ABTS^{•+}$ + Se/N-CQDs mixed solutions S1, respectively. The UV-Vis absorption spectra were recorded by a UV-3600 UV-VIS-NIR spectrophotometer under the same conditions. All solutions were prepared in ultra-pure water at room temperature.

S1.6. Effect of pH on the ability of Se/N-CQDs to scavenge $ABTS^{•+}$

To reserach the effect of pH on the ability of Se/N-CQDs to scavenge $ABTS^{•+}$, excess Se/N-CQDs were added into $ABTS^{•+}$ firstly. 250 μL ultra-pure

water and various aqueous solutions with different pH (0.78, 1.82, 2.60, 3.46, 4.23, 5.10, 6.03, 7.25, 8.36, 9.36, 10.35, 11.16, 12.03, 12.95 and 13.55) were added into 800 μL $ABTS^{•+}$ ($A_{734\ nm}$ = 0.70) and 50 μL Se/N-CQDs (1 mg/mL) mixed solutions (Se/N-CQDs + $ABTS^{•+}$ mixed solutions S2), respectively.

Furthermore, when a small amount of Se/N-CQDs were added into $ABTS^{•+}$, the effect of pH on the ability of Se/N-CQDs to scavenge $ABTS^{•+}$ was also investigated. 250 μL ultra-pure water and various aqueous solutions with different pH (0.78, 1.82, 2.60, 3.46, 4.23, 5.10, 6.03, 7.25, 8.36, 9.36, 10.35, 11.16, 12.03, 12.95 and 13.55) were added into the 800 μL Se/N-CQDs + $ABTS^{•+}$ mixed solutions S1, respectively.

The influence of pH on absorbance of Se/N-CQDs or $ABTS^{•+}$ were furthe studied, respectively. 3 mL ultra-pure water and various aqueous solutions with different pH (0.78, 1.82, 2.60, 3.46, 4.23, 5.10, 6.03, 7.25, 8.36, 9.36, 10.35, 11.16, 12.03, 12.95 and 13.55) were added into the 1 mL Se/N-CQDs (1 mg/mL) solutions at 734 nm under the same conditions, respectively. 250 μL ultra-pure water and various aqueous solutions with different pH (0.78, 1.82, 2.60, 3.46, 4.23, 5.10, 6.03, 7.25, 8.36, 9.36, 10.35, 11.16, 12.03, 12.95 and 13.55) were added into the 800 μL $ABTS^{•+}$ ($A_{734\ nm}$ = 0.70) solutions, respectively.

The UV-Vis absorption spectra were recorded by a UV-3600 UV-VIS-NIR spectrophotometer under the same conditions. All solutions were prepared in ultra-pure water at room temperature.

S2. Results and discussion

S2.1. Figures and tables

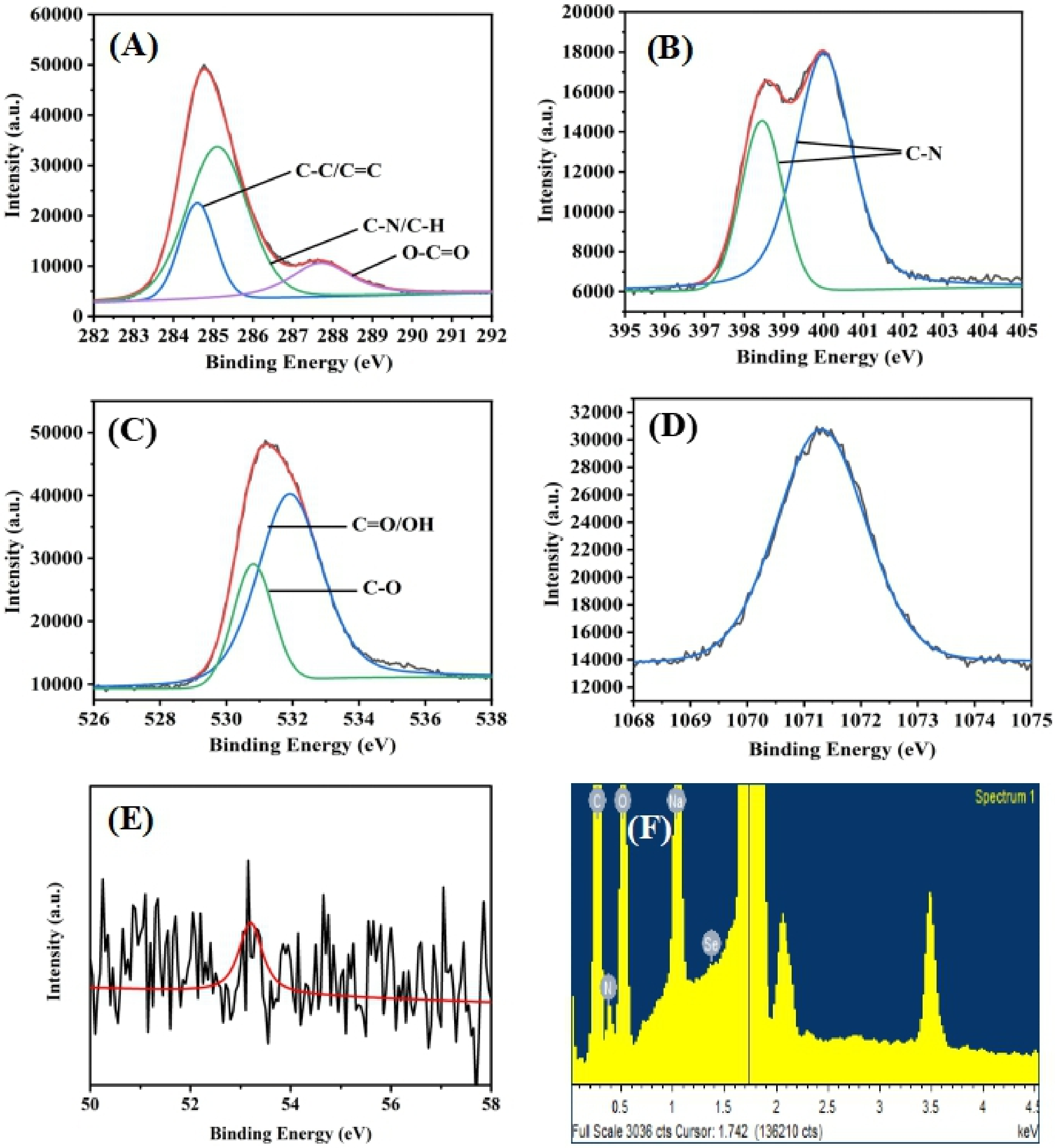

Fig. S1. (A) High-resolution XPS spectrum of C_{1s}. (B) High-resolution XPS spectrum of N_{1s}. (C) High-resolution XPS spectrum of O_{1s}. (D) High-resolution XPS spectrum of Na_{1s}. (E) High-resolution XPS spectrum of Se_{3d}. (F) EDS spectrum of Se/N-CQDs.

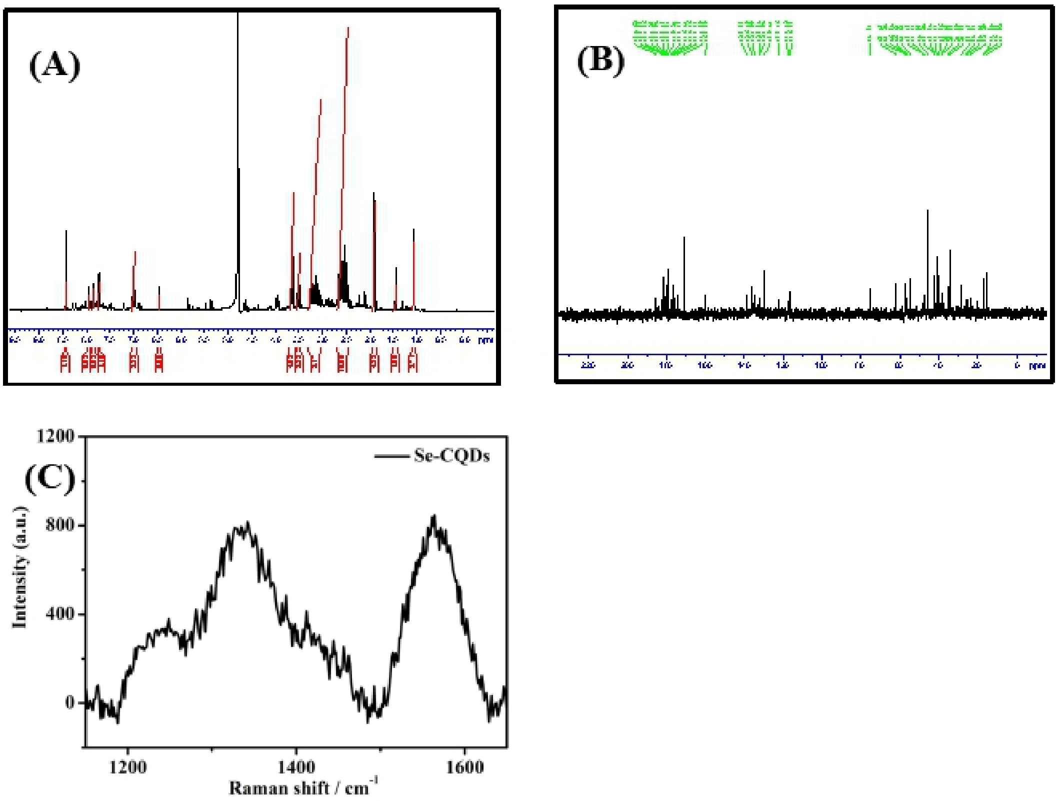

Fig. S2. (A) ^{1}H NMR spectrum of Se/N-CQDs. (B) ^{13}C NMR spectrum of Se/N-CQDs. (C) Raman spectrum of Se/N-CQDs.

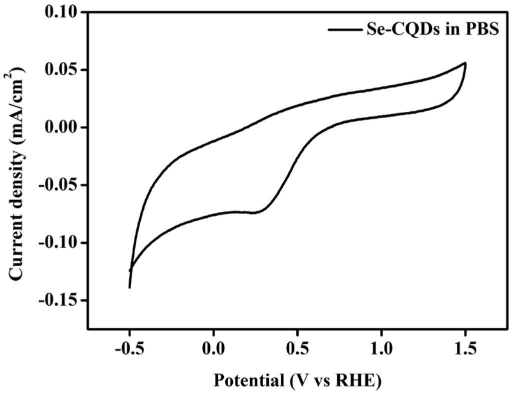

Fig. S3. The CV curve of Se/N-CQDs in phosphate buffer (pH = 7.20) with the scan range of -0.5 – 1.5 V at the rate of 100 mV/s.

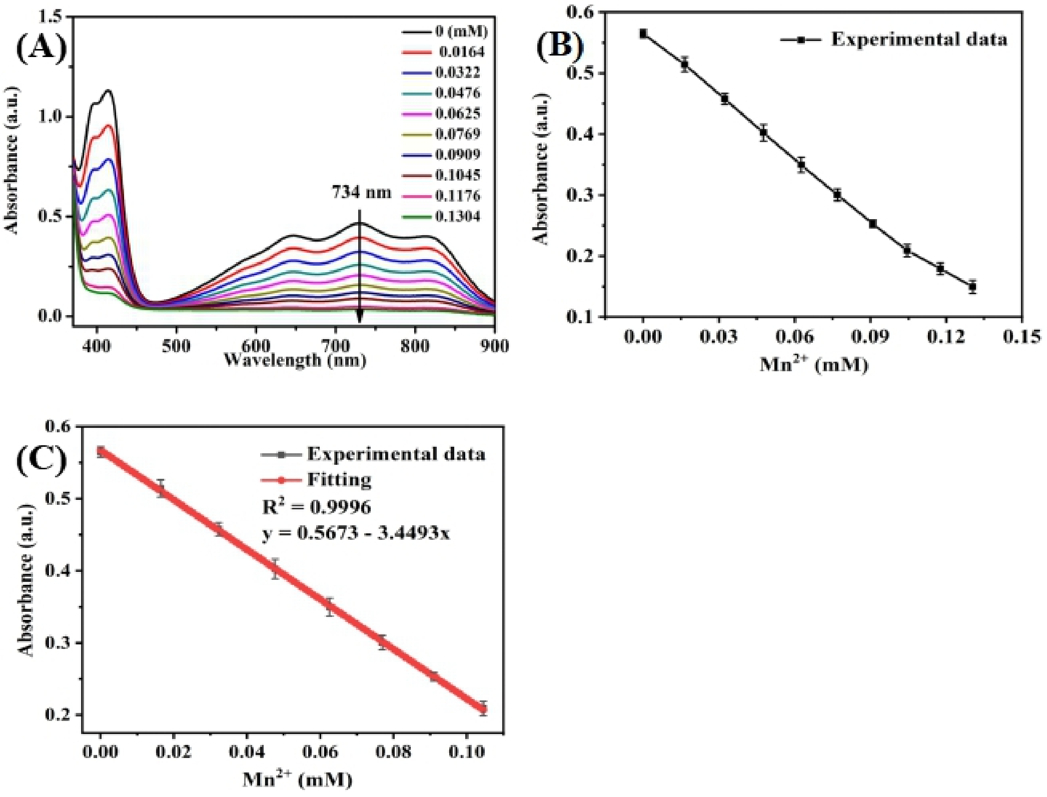

Fig. S4. (A) The UV-Vis absorption spectra of Se/N-CQDs + $ABTS^{\bullet+}$ mixed solutions (Mixed solutions) containing different concentrations of Mn(II) in tap water. (B) The UV-Vis absorbance of Mixed solutions containing different concentrations of Mn(II) in tap water from 0 to 130.40 μM at 734 nm. (C) The linear relationship between the UV-Vis absorbance of Mixed solutions and the concentrations of Mn(II) in tap water from 0 to 104.50 μM at 734 nm.

S2.2. Characterizations of Se/N-CQDs

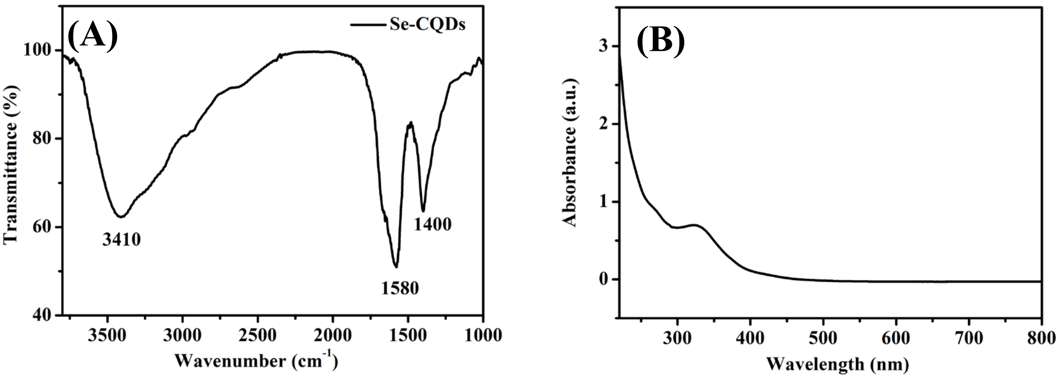

Fig. S5. (A) FTIR spectrum of Se/N-CQDs. (B) UV-Vis spectrum of Se/N-CQDs.

The FTIR spectrum of Se/N-CQDs was shown in Fig. S5A. The band at 3410 cm^{-1} was the stretching vibration of O-H and N-H bonds [S2, S3]. The peak at 1580 cm^{-1} were attributed to stretching vibration of C=O bonds[S1, S2]. The band at 1400 cm^{-1} was ascribed to C-N stretching vibrations [S3]. The results of FTIR and XPS demonstrated that there were carbon-containing groups, oxygen-containing groups and nitrogen-containing groups on the surface of Se/N-CQDs, which were very important for the formation of CQDs and had an effect on the fluorescence intensity of Se/N-CQDs [S4]. Fig. S5B was the UV-Vis spectrum of Se/N-CQDs. The weak absorbance peak at 326 nm was attributed to the n-π^* transitions of the carboxyl groups (O-C=O) on the surface of Se/N-CQDs [S5].

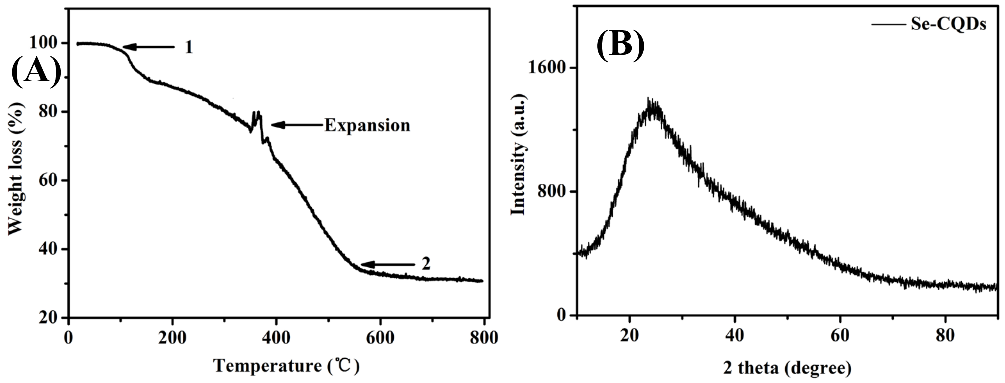

Fig. S6. (A) TGA spectrum of Se/N-CQDs. (B) XRD pattern of Se/N-CQDs.

The thermal stability of Se/N-CQDs was demonstrated by the TGA curve in Fig. S6A. The thermogram exhibited a two-step degradation pattern. Due to the elimination of water molecules or the moisture associated with Se/N-CQDs, the initial quality was reduced by 2% at 100 °C, illustrating that the thermal stability of Se/N-CQDs was up to 100 °C. A jump was attributed to the expansion of Se/N-CQDs between 350 and 390 °C. Owing to the gradual degradation of the surface functional groups of Se/N-CQDs, the final degradation step resulted in a significant weight loss (70%) in the range of 100–560 °C [S6]. The curve tended to be stable beyond 560 °C. Fig. S6B was XRD pattern of Se/N-CQDs. It could be seen that the broad X-ray diffraction peak at about 2θ = 24.9 was the characteristic graphitic carbon peak (PDF#74-2329), which further demonstrated the formation of Se/N-CQDs [S7].

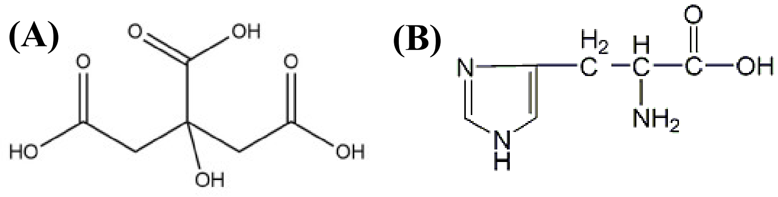

Fig. S7. The chemical structures of (A) citric acid and (B) L-Histidine.

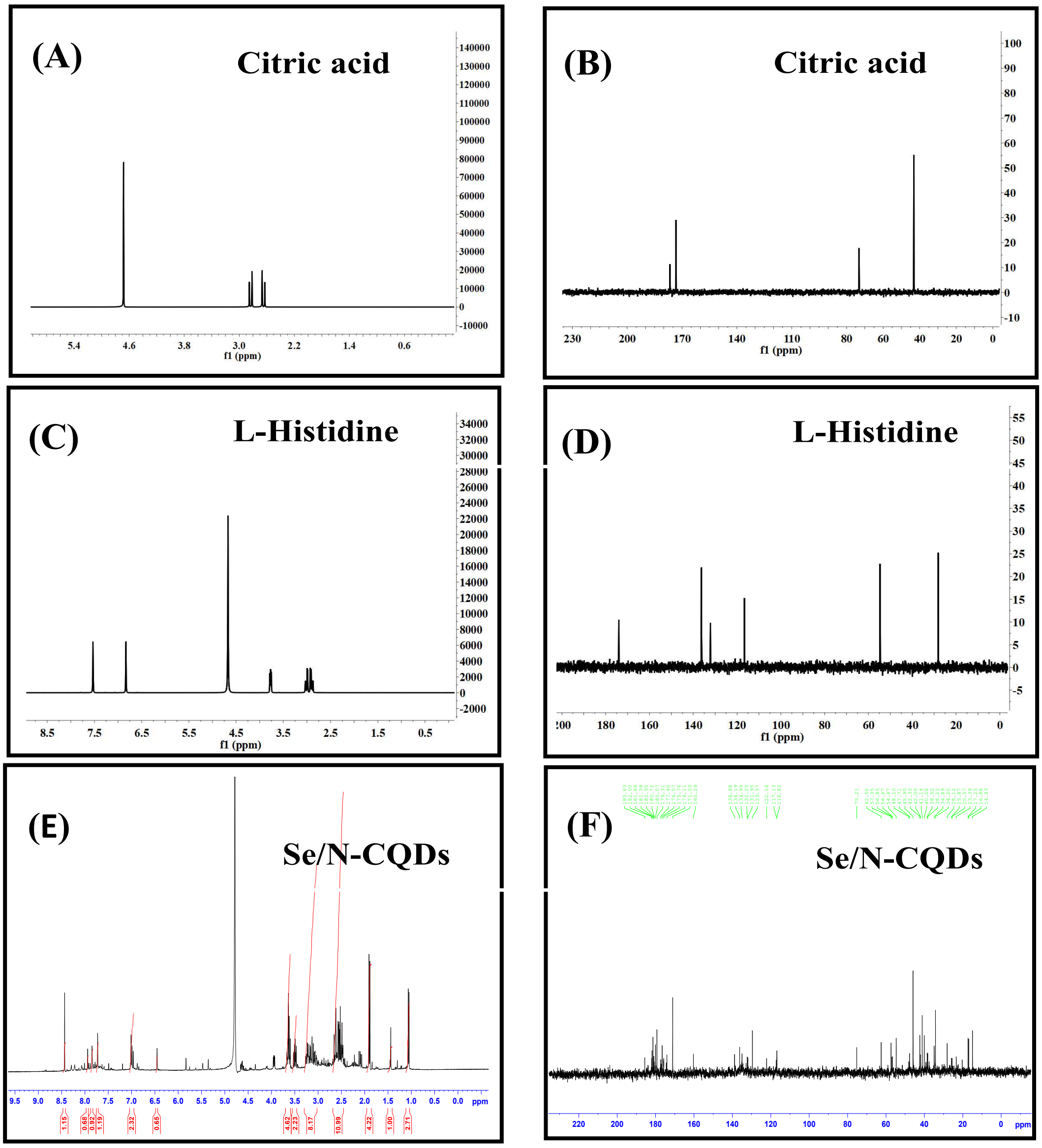

Fig. S8. (A) ^{1}H NMR spectrum of citric acid. (B) ^{13}C NMR spectrum of citric acid. (C) ^{1}H NMR spectrum of L-Histidine. (D) ^{13}C NMR spectrum of L-Histidine. (E) ^{1}H NMR spectrum of Se/N-CQDs. (F) ^{13}C NMR spectrum of Se/N-CQDs.

^{1}H NMR and ^{13}C NMR spectra of citric acid, L-Histidine and Se/N-CQDs were recorded using a Bruker 600 MHz, the chemical shifts were reported as the delta scale in ppm relative to D2O (δ = 4.79 ppm) for ^{1}H NMR. In the ^{1}H

NMR spectra, sp^2 hybridized carbon atoms signals were found in the range of 7–9 ppm, suggesting the aromatic structures in CQDs [S8, S9]. In the ^{13}C NMR spectra, signals detected in the range of 15–70 ppm and 100–185 ppm correspond to aliphatic (sp^3) carbon atoms and sp^2 hybridized carbon atoms, respectively. Signals between 170–185 ppm should correspond to carboxyl/ amide groups on the surface of CQDs [S8–S12]. Fig. S8E was the ^1H NMR spectrum of Se/N-CQDs. Signals between 6 ppm and 9 ppm belong to sp^2 hybridized carbon atoms, illustrating the aromatic structures of Se/N-CQDs [S9]. ^{13}C NMR spectrum of Se/N-CQDs was shown in Fig. S8F, signals between 25 ppm and 70 ppm were attributable to aliphatic (sp^3) carbon atoms. Signals between 100 ppm and 180 ppm were ascribed to sp^2 hybridized carbon atoms [S9, S13]. Especially, signals between 160 ppm and 180 ppm should be put down to carboxy/amide groups on the surface of Se/N-CQDs [S9]. From the ^1H NMR and ^{13}C NMR of spectra in Fig S7 and S8, compared with citric acid and L-Histidine, aliphatic (sp^3) carbon atoms, sp^2 hybridized carbon atoms and carboxyl/amide groups were retained in the Se/N-CQDs. Compared with other reported CQDs[S8–S18],the NMR results of Se/N-CQDs had almost no change. There were also aliphatic (sp^3) carbon atoms, sp^2 hybridized carbon atoms and carboxyl/amide groups in the CQDs [S8–S18].

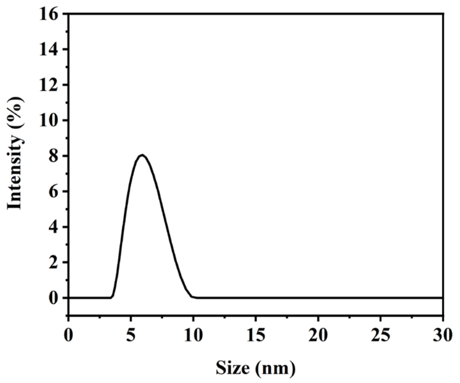

Fig. S9. The size distribution of Se/N-CQDs by DLS intensity.

The Zeta-potential and size distribution of Se/N-CQDs were measured by using the Nano-ZS90 zetasizer (Malvern Instruments Corp, UK.). The Zeta potential of Se/N-CQDs was -13.8 mV in phosphate buffer (pH = 7.4, 0.01 M). As shown in Fig. S9, the size distribution of Se/N-CQDs was about 6 ± 2 nm.

Tab. S5. XRF analysis of Se/N-CQDs.

Formula	Concentration (%)
CO_2	81.3
Na_2O	18.2
SeO_2	0.471
SiO_2	0.0074
SO_3	0.0108
K_2O	0.0129
ZnO	0.0022

The concentration (%) of Se in the powder sample was analyzed by X-ray fluorescence (XRF, Rigaku, XRF primus-2, Japan) and summarized in Tab. R1. The concentration (%) of Se as SeO_2 in the powder sample was 0.471%. It was

characterized by elemental analyzer (Elementar, vario El cube, German), the content of N was 9.32% in the 2.359 g powder sample (Se/N-CQDs).

S2.3. Study on the fluorescence intensity of Se/N-CQDs

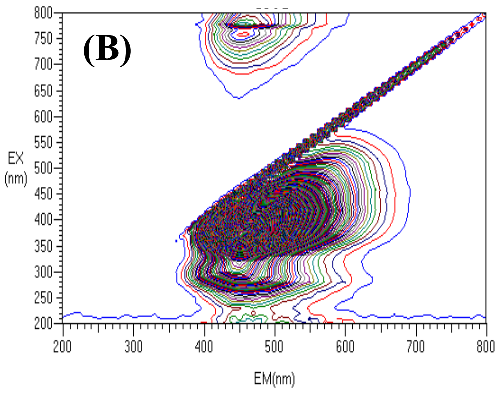

Fig. S10 The 3-D fluorescence spectrum of Se/N-CQDs.

The 3-D fluorescence spectrum of Se/N-CQDs was shown in Fig. S10. The excitation wavelength of Se/N-CQDs was about 250 to 550 nm.The emission wavelength of Se/N-CQDs was about 380 to 650 nm.

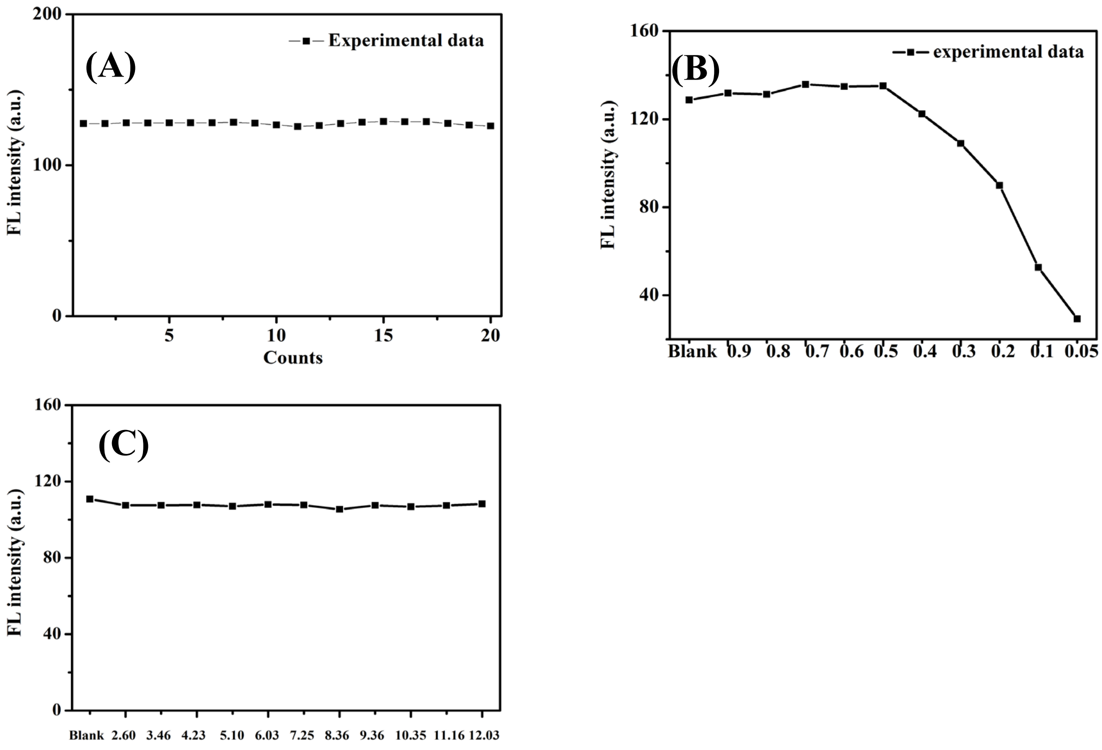

Fig. S11. (A) The maximum fluorescence intensity obtained by continuously measuring the fluorescence intensity of Se/N-CQDs, λ ex = 370 nm. (B) The maximum fluorescence intensity of Se/N-CQDs (1 mg/mL) diluted by PBS (pH = 7.40, 0.01 M) with different dilutions (0.9, 0.8, 0.7, 0.6, 0.5, 0.4, 0.3, 0.2, 0.1 and 0.05). (C) The maximum fluorescence intensity of Se/N-CQDs containing aqueous solutions at different pH (2.60, 3.46, 4.23, 5.10, 6.03, 7.25, 8.36, 9.36, 10.35, 11.16, 12.03) .

The fluorescence intensity of Se/N-CQDs were continuously measured for 20 counts when the excitation wavelength was 370 nm. As shown in Fig. S11A, the maximum fluorescence intensities were almost the same (RSD = 8.51%), showing that the fluorescent probe based on Se/N-CQDs had good stability. When Se/N-CQDs (1 mg/mL) were diluted to 0.9, 0.8, 0.7, 0.6, 0.5, 0.4, 0.3, 0.2, 0.1 and 0.05 times, it can be seen from Fig. S11B that the fluorescence intensities increased slowly at the beginning, the fluorescence intensity was maximum when Se/N-CQDs (1 mg/mL) were diluted to 0.5 time. Then, the fluorescence intensities decreased gradually. This phenomenon in fluorescence intensities might be related to self-quenching which occurred at high concentration due to the aggregation quenching effect. The effect of pH (2.60, 3.46, 4.23, 5.10, 6.03, 7.25, 8.36, 9.36, 10.35, 11.16 and 12.03) on the fluorescence intensity of Se/N-CQDs was further discussed. As shown in Fig. S11C, the fluorescence intensities of mixed solutions at different pH were almost unchanged, indicating that pH has no effect on the fluorescence intensity of Se/N-CQDs.

S2.4. Effect of ionic strength on the ability of Se/N-CQDs to scavenge ABTS$^{\bullet+}$

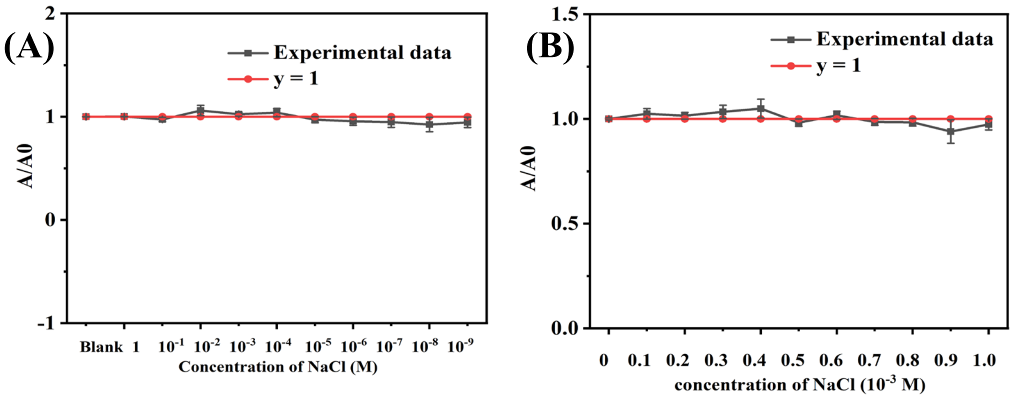

Fig. S12. (A) The relationship between A/A0 and the concentration of NaCl, when different concentrations of NaCl with the different orders of magnitude were added into the Se/N-CQDs + ABTS$^{\bullet+}$ mixed solutions S1 at 734 nm. (B) The relationship between A/A0 and the concentration of NaCl at 734 nm, when different concentrations of NaCl with the same order of magnitude were added into the ABTS$^{\bullet+}$ + Se/N-CQDs mixed solutions S1 at 734 nm.

To confirm the ability of Se/N-CQDs to scavenge ABTS$^{\bullet+}$ free radical under ionic strength environments, different concentrations of NaCl with the different orders of magnitude and the same order of magnitude were added into the Se/N-CQDs + ABTS$^{\bullet+}$ mixed solutions S1 at 734 nm under the same conditions. It could be seen from Fig. S12A and S9B that the lines were almost straight, some concentrations of NaCl caused slight changes in the absorbance of Se/N-CQDs + ABTS$^{\bullet+}$, but these effects might be negligible, showing that ionic strength had almost no effect on the ability of Se/N-CQDs to scavenge ABTS$^{\bullet+}$.

S2.5. Effect of pH on the ability of Se/N-CQDs to scavenge $ABTS^{\bullet+}$

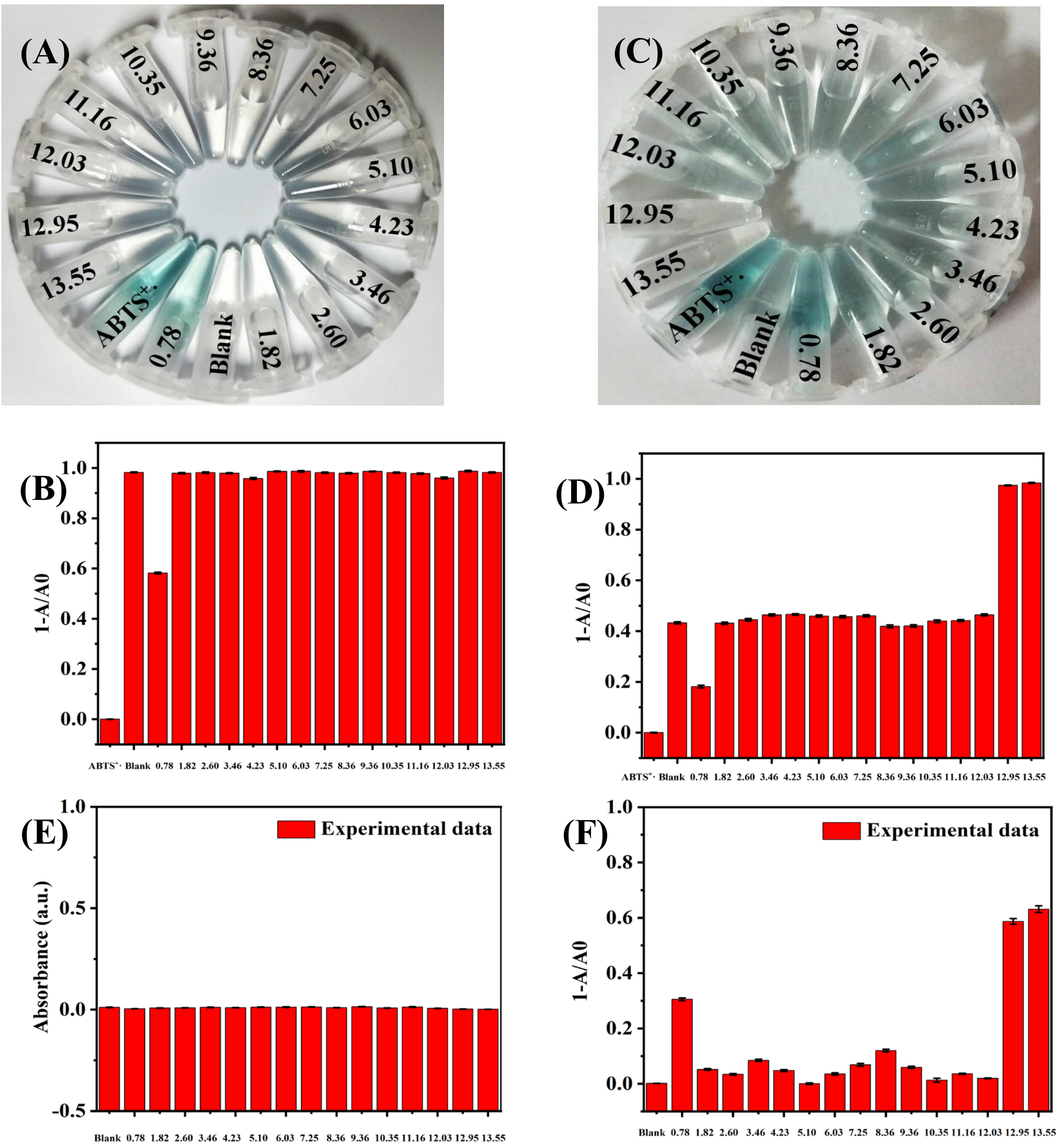

Fig. S13. (A) Photographs of $ABTS^{\bullet+}$ and Se/N-CQDs + $ABTS^{\bullet+}$ mixed solutions S2 (excess Se/N-CQDs were added) containing water and various aqueous solutions at different pH. (B) The relationship between 1-A/A0 and pH at 734 nm. A was the UV-Vis absorbance of Se/N-CQDs + $ABTS^{\bullet+}$ mixed solutions S2 (excess Se/N-CQDs were added) containing water and various aqueous solutions at different pH. A0 was the UV-Vis absorbance of $ABTS^{\bullet+}$. (C) Photographs of $ABTS^{\bullet+}$ and Se/N-CQDs + $ABTS^{\bullet+}$ mixed solutions S1 (a

small amount of Se/N-CQDs were added) which containing water and various aqueous solutions at different pH. (D) The relationship between 1-A/A0 and pH at 734 nm. A was the UV-Vis absorbance of Se/N-CQDs + $ABTS^{\bullet+}$ mixed solutions S1 (a small amount of Se/N-CQDs were added) containing water and various aqueous solutions at different pH. A0 was the UV-Vis absorbance of $ABTS^{\bullet+}$. (E) The UV-Vis absorbance of Se/N-CQDs containing aqueous solutions at different pH at 734 nm. (F) The relationship between 1-A/A0 and pH at 734 nm. A was the UV-Vis absorbance of $ABTS^{\bullet+}$ solutions containing various aqueous solutions at different pH. A0 was the UV-Vis absorbance of $ABTS^{\bullet+}$ solutions containing water.

It could be seen directly from Fig. S13A and S10B, when water (blank) and various aqueous solutions at different pH (0.78, 1.82, 2.60, 3.46, 4.23, 5.10, 6.03, 7.25, 8.36, 9.36, 10.35, 11.16, 12.03, 12.95 and 13.55) were added into Se/N-CQDs + $ABTS^{\bullet+}$ mixed solutions S2 which excess Se/N-CQDs were added. $ABTS^{\bullet+}$ could be completely scavenged at pH 1.82, 2.60, 3.46, 4.23, 5.10, 6.03, 7.25, 8.36, 9.36, 10.35, 11.16, 12.03, 12.95 and 13.55. However, the content of $ABTS^{\bullet+}$ increased under the strong acid condition at pH 0.78. Due to the positive charge of $ABTS^{\bullet+}$, strong acidic conditions might be beneficial to the formation and stability of $ABTS^{\bullet+}$. When water (blank) and various aqueous solutions at different pH (0.78, 1.82, 2.60, 3.46, 4.23, 5.10, 6.03, 7.25, 8.36, 9.36, 10.35, 11.16, 12.03, 12.95 and 13.55) were added into Se/N-CQDs + $ABTS^{\bullet+}$ mixed solutions S1 which a small amount of Se/N-CQDs were added.

As shown in Fig. S13C and S10D, pH (1.82, 2.60, 3.46, 4.23, 5.10, 6.03, 7.25, 8.36, 9.36, 10.35, 11.16 and 12.03) could hardly affect the ability of Se/N-CQDs to scavenge $ABTS^{•+}$. However, the content of $ABTS^{•+}$ increased under the strong acid condition at pH 0.78, $ABTS^{•+}$ could be completely scavenged at pH 12.95 and 13.55. Therefore, pH affected the ability of Se/N-CQDs to scavenge $ABTS^{•+}$ under strong basic conditions and under the strong acid condition, and pH could hardly affect the ability of Se/N-CQDs to scavenge $ABTS^{•+}$ under mild conditions. As seen from the Fig. S13E, Se/N-CQDs had almost no absorbance when aqueous solutions with different pH were added into Se/N-CQDs at 734 nm, indicating that Se/N-CQDs might not affect the UV-Vis absorbance of $ABTS^{•+}$ at 734 nm. However, pH could affect the absorbance of $ABTS^{•+}$ at 734 nm in Fig. S13F. The results of pH affecting $ABTS^{•+}$ were consistent with the results of pH affecting the ability of Se/N-CQDs to scavenge $ABTS^{•+}$. In short, the impact of pH on the ability of Se/N-CQDs scavenging $ABTS^{•+}$ was due to the direct impact of pH on $ABTS^{•+}$. pH would not affect the ability of Se/N-CQDs scavenging $ABTS^{•+}$ in neutral, weak acid and weak alkaline environments. This was conducive to the application of Se/N-CQDs as an antioxidant in biomedicine and other fields.

S2.6. Stability of Se/N-CQDs + $ABTS^{•+}$ mixed solutions 1 for Mn(II) detection

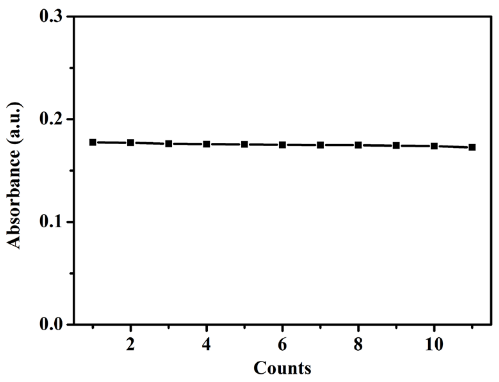

Fig. S14. The absorbance of Se/N-CQDs + $ABTS^{\bullet+}$ mixed solutions 1 when it was continuously measured at 734 nm.

The absorbance of Se/N-CQDs + $ABTS^{\bullet+}$ mixed solutions 1 were continuously measured for 11 counts in 15 minutes. As shown in Fig. S14, the absorbance at 734 nm were almost the same (RSD = 0.808%), showing that Se/N-CQDs + $ABTS^{\bullet+}$ mixed solutions also had excellent stability.

S2.7. Experimental conditions of the colorimetric sensor based on Se/N-CQDs + $ABTS^{\bullet+}$ for the optimized assay for Mn(II) detection.

Some experimental conditions like the ratio of $ABTS^{\bullet+}$ and Se/N-CQDs, the reaction time between the colorimetric sensor and Mn(II) were investigated for the optimized assay for Mn(II) detection. Eight groups of experiments on the ratio of $ABTS^{\bullet+}$ and Se/N-CQDs were shown in Tab. S6. Two groups of experiments on the reaction time between the colorimetric sensor based on the $ABTS^{\bullet+}$ and Se/N-CQDs and Mn(II) were shown in Tab. S7. The UV-Vis absorption spectra of $ABTS^{\bullet+}$ (2 mL, $A_{734\ nm}$ = 0.7) + Se/N-CQDs (25 μL, 1 mg/mL) + Mn(II) (100 μL, 1 mM) and $ABTS^{\bullet+}$ (2 mL, $A_{734\ nm}$ = 0.7) + Se/N-CQDs (25 μL, 1 mg/mL) + Mn(II) (200 μL, 1 mM) were measured for 30

times in 3min.

Tab. S6. Eight groups of experiments on the ratio of $ABTS^{•+}$ and Se/N-CQDs.

	$ABTS^{•+}$ ($A_{734\ nm}$ = 0.7, μL)	Se/N-CQDs (1 mg/mL, μL)	Water (μL)
1	2000	0	400
2	2000	25	375
3	2000	50	350
4	2000	75	325
5	2000	100	300
6	2000	200	200
7	2000	300	100
8	2000	400	0

Tab. S7. Two groups of experiments on the reaction time between the colorimetric sensor and Mn(II).

	$ABTS^{•+}$ ($A_{734\ nm}$ = 0.7, μL)	Se/N-CQDs (1 mg/mL, μL)	Mn(II) (1 mM, μL)	Water (μL)
1	2000	25	100	200
2	2000	25	200	100

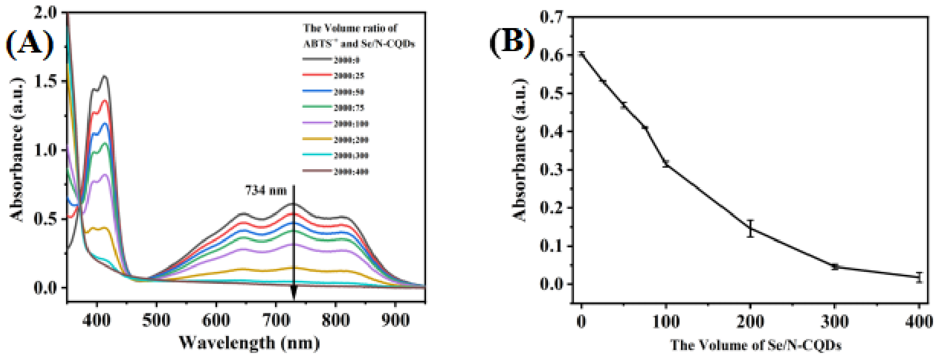

Fig. S15. (A) The UV-Vis absorption spectra of $ABTS^{\bullet+}$ (2 mL, $A_{734\ nm} = 0.7$) in the presence of different Volume of Se/N-CQDs (0, 25, 50, 75, 100, 200, 300 and 400 μL, 1 mg/mL). (B) The absorbance at 734 nm of $ABTS^{\bullet+}$ (2 mL, $A_{734\ nm} = 0.7$) in the presence of different Volume of Se/N-CQDs (0, 25, 50, 75, 100, 200, 300 and 400 μL, 1 mg/mL).

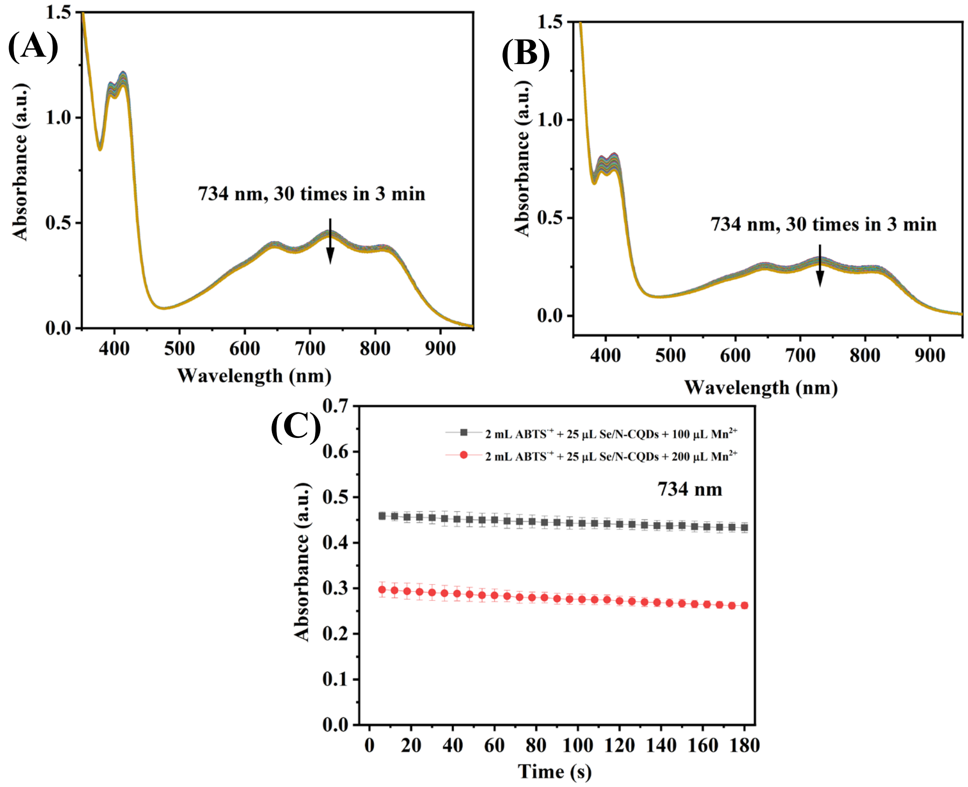

Fig. S16. (A) The UV-Vis absorption spectra of $ABTS^{\bullet+}$ (2 mL, $A_{734\ nm} = 0.7$) + Se/N-CQDs (25 μL, 1 mg/mL) + Mn^{2+} (100 μL, 1 mM) for 30 times in 3min. (A) The UV-Vis absorption spectra of $ABTS^{\bullet+}$ (2 mL, $A_{734\ nm} = 0.7$) +

Se/N-CQDs (25 μL, 1 mg/mL) + Mn^{2+} (200 μL, 1 mM) for 30 times in 3min. (C) The absorbance at 734 nm of $ABTS^{•+}$ (2 mL, $A_{734\ nm}$ = 0.7) + Se/N-CQDs (25 μL, 1 mg/mL) + Mn^{2+} (100 μL, 1 mM) and $ABTS^{•+}$ (2 mL, $A_{734\ nm}$ = 0.7) + Se/N-CQDs (25 μL, 1 mg/mL) + Mn^{2+} (200 μL, 1 mM) for 30 times in 3min.

As shown in Fig. S15, when the different volumes of Se/N-CQDs were added into $ABTS^{•+}$, respectively, $ABTS^{•+}$ was scavenged with the increase of Se/N-CQDs. Moreover, based on the synergistic effect of Se/N-CQDs and Mn(II) on $ABTS^{•+}$, Se/N-CQDs and $ABTS^{•+}$ as the colorimetric sensor was applied to the detection of Mn(II). Thereby, a small amount of Se/N-CQDs were added into $ABTS^{•+}$ to form a colorimetric sensor for detecting Mn(II). A part of $ABTS^{•+}$ was reserved and Se/N-CQDs and Mn(II) had the synergistic effect to scavenge $ABTS^{•+}$ for the Mn(II) detection. When 25 μL of Se/N-CQDs were added into 2 mL of $ABTS^{•+}$, $ABTS^{•+}$ could be scavenged, so Se/N-CQDs + $ABTS^{•+}$ mixed solutions (Mixed solutions) were prepared by 40 mL $ABTS^{•+}$ ($A_{734\ nm}$ = 0.70) and 500 μL Se/N-CQDs (1 mg/mL) in the manuscript.

As shown in Fig. S16, when the UV-Vis absorption spectra of $ABTS^{•+}$ (2 mL, $A_{734\ nm}$ = 0.7) + Se/N-CQDs (25 μL, 1 mg/mL) + Mn(II) (100 μL, 1 mM) and $ABTS^{•+}$ (2 mL, $A_{734\ nm}$ = 0.7) + Se/N-CQDs (25 μL, 1 mg/mL) + Mn(II) (200 μL, 1 mM) were measured for 30 times in 3min, the redox reaction occurred rapidly, with the increase of reaction time, the absorbance at 734 nm had a slight negligible decrease. Therefore, the absorbance was measured

immediately after the sample was prepared.

S2.8. The reproducibility of the colorimetric sensor based on $ABTS^{•+}$ + Se/N-CQDs for the detection of Mn(II).

Tab. S8. Three groups of experiments on the reproducibility of the colorimetric sensor based on $ABTS^{•+}$ + Se/N-CQDs for the Mn(II) detection.

$ABTS^{•+}$ (μL)	Se/N-CQDs (μL)	Mn(II) (μL)	K2S2O8(μL)	Water (μL)
2000	0	0	0	1500
2000	100	400	0	1000
2000	100	400	1000	0

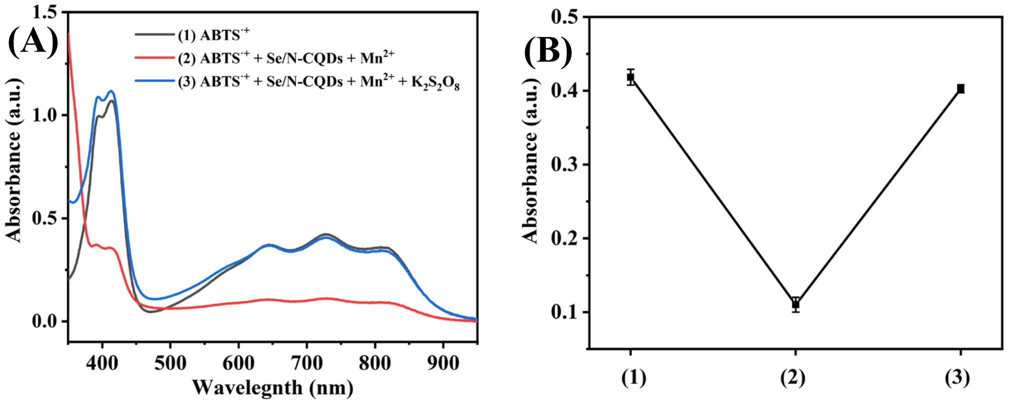

Fig. S17. (A) The UV-Vis absorption spectra of $ABTS^{•+}$, $ABTS^{•+}$ + Se/N-CQDs + Mn^{2+}, $ABTS^{•+}$ + Se/N-CQDs + Mn^{2+} + $K_2S_2O_8$. (B) The absorbance at 734 nm of $ABTS^{•+}$, $ABTS^{•+}$ + Se/N-CQDs + Mn^{2+}, $ABTS^{•+}$ + Se/N-CQDs + Mn^{2+} + $K_2S_2O_8$.

To study the reproducibility of the colorimetric sensor based on $ABTS^{•+}$ + Se/N-CQDs for the detection of Mn(II), $K_2S_2O_8$ was used to investigate the reproducibility of the colorimetric sensor based on $ABTS^{•+}$ + Se/N-CQDs. Three groups of experiments on the reproducibility of the colorimetric sensor based on

$ABTS^{\bullet+}$ + Se/N-CQDs for the Mn(II) detection were shown in Tab. S8. As shown in Fig. S17, when $K_2S_2O_8$ was added into $ABTS^{\bullet+}$ + Se/N-CQDs + Mn(II), the absorbance at 734 nm increased significantly and was similar to that of $ABTS^{\bullet+}$, showing that $ABTS^{\bullet+}$ increased and recovered in $ABTS^{\bullet+}$ + Se/N-CQDs + Mn(II) + $K_2S_2O_8$ solutions. Therefore, when moderate Se-CQDs were added into the $ABTS^{\bullet+}$ + Se/N-CQDs + Mn(II) + $K_2S_2O_8$ solutions again, the mixed solutions might be used for the Mn(II) detection again. The colorimetric sensor based on $ABTS^{\bullet+}$ and Se/N-CQDs could be recycled for the Mn(II) detection and be a reproducible colorimetric sensor.

S2.8. Tables

Tab. S1. Four groups of the synergy of Mn(II) and Se/N-CQDs.

	$ABTS^{\bullet+}$ (mL)	Water (μL)	Mn(II) (1 mM, μL)	Se/N-CQDs (1 mg/mL, μL)
1	2	300	0	0
2	2	50	250	0
3	2	250	0	50
4	2	0	250	50

Tab. S2. Comparison of different probes for the detection of Mn(II).

Detection probe	LOD (μM)	Ref.
Multifunctional sensor	6.03	Lee et al. [S19]
Silver nanoparticles	1.70	Mehta et al. [S20]
Schiff base chemosensor	5.0	Hariharan et al. [S21]
Schiff base	7.11	Kim et al. [S22]
Copper nanoclusters	10	Han et al. [S23]
Se/N-CQDs	1.69	This work

Tab. S3. Analysis of Mn(II) in ultra-pure water determined by the colorimetric probe based on Se/N-CQDs + $ABTS^{•+}$ method.

Sample	Added Mn(II) (μM)	Found (μM)	Recovery (%, n = 3)	RSD/%
1	62.50	66.00	105.60	8.27
2	90.90	95.90	105.50	5.95
3	117.60	122.00	103.70	3.15
4	142.90	138.00	96.60	7.52

Tab. S4. Analysis of Mn(II) in tap water determined by the colorimetric probe based on $ABTS^{•+}$ + Se/N-CQDs and ICP-OES methods.

Sample	Added Mn(II) (μM)	$ABTS^{•+}$ + Se/N-CQDs Found (μM)	Recovery (%, n = 3)	RSD/%	ICP-OES Found (μM)	Recovery (%, n = 3)	RSD/%
1	32.20	33.30	103.42	3.55	31.87	98.98	2.20
2	47.60	49.44	103.86	2.28	48.27	101.41	1.25
3	62.50	63.00	100.80	8.14	62.13	99.41	1.61
4	76.90	76.42	99.38	11.35	76.43	99.39	0.89

S3. References

[S1] R.Z. Zhang, W. Chen, Nitrogen-doped carbon quantum dots: Facile synthesis and application as a "turn-off" fluorescent probe for detection of Hg^{2+} ions. Biosensors and Bioelectronics 2014, 55, 83–90.

[S2] J. Shen, S.M. Shang, X.Y. Chen, D. Wang, Y. Cai, Highly fluorescent N, S-co-doped carbon dots and their potential applications as antioxidants and sensitive probes for Cr (VI) detection. Sensors and Actuators B: Chemical 2017, 248, 92–100.

[S3] Z.S. Qian, X.Y. Shan, L.J. Chai, J.J. Ma, J.R. Chen, H. Feng, DNA nanosensor based on biocompatible graphene quantum dots and carbon nanotubes. Biosensors and Bioelectronics 2014, 60, 64–70.

[S4] S.J. Zhu, Q.N. Meng, L. Wang, J.H. Zhang, Y.B. Song, H. Jin, K. Zhang, H.C. Sun, H.Y. Wang, B. Yang, Highly photoluminescent carbon dots for multicolor patterning, sensors, and bioimaging. Angewandte Chemie International Edition 2013, 125, 4045–4049.

[S5] H.K. Sadhanala, K.K. Nanda, Boron-doped carbon nanoparticles: Size-independent color tunability from red to blue and bioimaging applications. Carbon 2016, 96, 166–173.

[S6] Sachdev, P. Gopinath, Green synthesis of multifunctional carbon dots from coriander leaves and their potential application as antioxidants, sensors and bioimaging agents. Analyst 2015, 140, 4260–4269.

[S7] Liu, T., Li, N., Dong, J.X., Zhang, Y., Fan, Y.Z., Lin, S.M., Li, N.B., A colorimetric and fluorometric dual-signal sensor for arginine detection by inhibiting the growth of gold nanoparticles/carbon quantum dots composite. Biosensors and Bioelectronics 2017, 87, 772–778.

[S8] C. Huang, H. Dong, Y. Su, et al. Synthesis of Carbon Quantum Dot Nanoparticles Derived from Byproducts in Bio-Refinery Process for Cell Imaging and In Vivo Bioimaging. Nanomaterials 2019, 9, 387.

[S9] F. Li, T.Y. Li, C.X. Sun, J.H. Xia, Y. Jiao, H.P. Xu, Selenium-Doped Carbon Quantum Dots for Free-Radical Scavenging. Angewandte Chemie International Edition 2017, 129, 10042–10046.

[S10] Z. Wang, F.L. Yuan, et al. 53% Efficient Red Emissive Carbon Quantum Dots for High Color Rendering and Stable Warm White-Light-Emitting Diodes. Advanced materials 2017, 1702910.

[S11] I. Singh, R. Arora, H. Dhiman, et al. Carbon Quantum Dots: Synthesis, Characterization and Biomedical Applications. Turkish Journal of Pharmaceutical Sciences 2018, 15, 219–230.

[S12] F.S. Wu, H.F. Su, et al. Facile synthesis of N-rich carbon quantum dots from porphyrins as efficient probes for bioimaging and biosensing in living cells. International Journal of Nanomedicine 2017, 12, 7375–7391.

[S13] K. Bankoti, A.P. Rameshbabu, S. Datta, B. Das, A. Mitra, S. Dhara, Onion derived carbon nanodots for live cell imaging and accelerated skin wound healing, Journal of Materials Chemistry B 2017, 5, 6579–6592.

[S14] J. Ye, K. Ni, J. Liu, et al. Oxygen-Rich Carbon Quantum Dots as Catalysts for Selective Oxidation of Amines and Alcohols. ChemCatChem, 2017, 10, 259–265.

[S15] C. Tan, S. Zuo, Y. Zhao, et al. Preparation of multicolored carbon quantum dots using $HNO_3/HClO_4$ oxidation of graphitized carbon. Journal of Materials Research

2019, 34, 1–11.

[S16] B.C.M. Martindale, G.A.M. Hutton, C.A. Caputo, et al. Solar Hydrogen Production Using Carbon Quantum Dots and a Molecular Nickel Catalyst. Journal of the American Chemical Society 2015, 137, 6018–6025.

[S17] P. Devi, T. Anupma, et al. Ultrasensitive and Selective Sensing of Selenium Using Nitrogen-Rich Ligand Interfaced Carbon Quantum Dots. Acs Appl.mater.interfaces 2017, 9, 15, 13448–13456

[S18] C.N. Virca, H.M. Winter, A.M. Goforth, et al. Photocatalytic water reduction using a polymer coated carbon quantum dot sensitizer and a nickel nanoparticle catalyst. Nanotechnology 2017, 28, 195402.

[S19] Y.J. Lee, C.J. Lim, H. Suh, E.J. Song, C. Kim, A multifunctional sensor: Chromogenic sensing for Mn^{2+} and fluorescent sensing for Zn^{2+} and Al^{3+}. Sensors and Actuators B: Chemical 2014, 201, 535–544.

[S20] V.N. Mehta, J.V. Rohit, S.K. Kailasa, Functionalization of silver nanoparticles with 5-sulfoanthranilic acid dithiocarbamate for selective colorimetric detection of Mn^{2+} and Cd^{2+} ions. New Journal of Chemistry 2016, 40, 4566–4574.

[S21] P.S. Hariharan, S.P. Anthony, Substitutional group dependent colori/fluorimetric sensing of Mn^{2+}, Fe^{3+} and Zn^{2+} ions by simple Schiff base chemosensor. Spectrochimica Acta Part A: Molecular and Biomolecular Spectroscopy 2015, 136, 1658–1665.

[S22] K.B. Kim, G.J. Park, H. Kim, E.J. Song, J.M. Bae, C. Kim, A novel colorimetric chemosensor for multiple target ions in aqueous solution: simultaneous detection of Mn(II) and Fe(II). Inorganic Chemistry Communications 2014, 46, 237–240.

[S23] B.Y. Han, R.C. Xiang, X.F. Hou, M.B. Yu, T.T. Peng, Y. Li, G.H. He, One-step rapid synthesis of single thymine-templated fluorescent copper nanoclusters for "turn on" detection of Mn^{2+}. Analytical Methods 2017, 9, 2590–2595.
